# Inulin-enriched *Megamonas funiformis* ameliorates metabolic dysfunction-associated fatty liver disease by producing propionic acid

**DOI:** 10.1038/s41522-023-00451-y

**Published:** 2023-11-04

**Authors:** Xinyue Yang, Meihong Zhang, Yan Liu, Fuxiao Wei, Xin Li, Yuqing Feng, Xiaolu Jin, Dan Liu, Yuming Guo, Yongfei Hu

**Affiliations:** State Key Laboratory of Animal Nutrition and Feeding, College of Animal Science and Technology, China Agricultural University, 100193 Beijing, China

**Keywords:** Microbiome, Metagenomics

## Abstract

Accumulated evidence supports the beneficial role of inulin in alleviating metabolic dysfunction-associated fatty liver disease (MAFLD) by modulating gut microbiota. However, the underlying mechanisms are not fully understood. Here we used high-fat diet (HFD)-induced laying hen model of MAFLD to investigate the effect of inulin on ameliorating MAFLD and found that the inulin-enriched *Megamonas* genus was inversely correlated with hepatic steatosis-related parameters. Oral administration of a newly isolated commensal bacterium by culturomics, *M. funiformis* CML154, to HFD-fed hens and mice ameliorated MAFLD, changed liver gene expression profiles, and increased intestinal propionate concentration. Further evidence demonstrated that the anti-MAFLD effect of *M. funiformis* CML154 is attributed to propionate-mediated activation of the *APN*-*AMPK*-*PPARα* signaling pathway, thereby inhibiting fatty acid de novo synthesis and promoting β-oxidation. These findings establish the causal relationships among inulin, *M. funiformis*, and MAFLD, and suggest that *M. funiformis* CML154 is a probiotic candidate for preventative or therapeutic intervention of MAFLD.

## Introduction

Metabolic associated fatty liver disease (MAFLD), previously known as nonalcohol fatty liver disease (NAFLD), is estimated to affect a quarter of population worldwide^1,2^. The incidence of MAFLD has increased rapidly in recent years, particular in youngers^1^, which is intimately associated to the high incidence of liver cirrhosis, diabetes, cardiovascular disease and various malignant tumors^2^. Currently, there is no approved pharmacological therapy for MAFLD other than lifestyle changes such as dietary interventions, weight loss and regular exercise^1^.

Increasing evidence has indicated that beneficial gut bacteria have anti-MAFLD properties. It is demonstrated that *Akkermansia muciniphila* relieves MAFLD by regulating the metabolism of L-aspartate via gut-liver axis^3^; *Desulfovibrio vulgaris* attenuates MAFLD via generating acetic acid^4^; and *Parabacteroides distasonis* alleviates obesity and metabolic dysfunctions via production of succinate and secondary bile acids^5^. Beyond these, *Bacteroides spp*, *F. prausnitzii*, and *Roseburia* all have been demonstrated to have the effect of alleviating MAFLD via different mechanisms^6^.

Inulin, a commonly used prebiotic, has been reported to modulate the gut microbiota and have various beneficial functions on the host, including improvement of endothelial dysfunction, prevention of metabolic disorders-related cardiovascular diseases, and regulation of lipid-metabolism in obese individuals^7^. The health-improving effects of inulin are mainly known to its role in promoting beneficial gut bacteria especially *Bifidobacterium* and/or *Lactobacillus* spp^8^. However, we previously found that inulin can enrich gut SCFA-producing *Prevotella* bacteria and thereby improves metabolic disorders in mice, suggesting that the prebiotic role of inulin may not be limited to traditionally recognized probiotics^9^. As the only accepted prebiotic that improved bowel function by European Food Safety Authority^8^, how inulin interacts with gut commensal microbes and thus benefit the host metabolisms still needs to be elucidated.

To better study the role of gut commensal microbes in MAFLD development and to develop novel therapeutic strategies, an ideal animal model is very necessary. Currently, diet-induced rodents and genetically modified mice are the most commonly used MAFLD animal models^10^. However, it is suggested that lipid metabolism in rodents is not the same as that in humans. For example, liver and adipose tissue in rodent models contribute equally to de novo lipogenesis^11^, but liver in humans bears more than 90% de novo lipogenesis^12^. Additionally, rodents have a lower incidence of MAFLD than humans during the natural processes of life. Interestingly, liver is the major site for de novo lipogenesis in chickens, which is very similar to that in humans, and excessive hepatic fat deposition and susceptibility to MAFLD are common pathological phenomena in laying hens during normal aging^12^. More and more studies proved that chicken, especially the laying hen, is an appealing animal used for understanding and investigating MAFLD in humans^12,13^.

In the present study, we found that inulin reduced body weight, fat accumulation and hepatic steatosis in a laying hen model of HFD-induced MAFLD by changing the gut microbiota composition, which was confirmed by an independent cohort of old laying hens. We isolated and identified an isolate named *M. funiformis* CML154 from the gut of the inulin-supplemented laying hens by culturomics. We demonstrated that the administration of strain CML154 causatively decreased HFD-induced MAFLD in both laying hen and mice models. We further confirmed that a specific metabolite of CML154, propionic acid, decreased lipid synthesis and promoted lipid oxidation in hepatocyte steatosis cell model.

## Results

### Inulin relieves MAFLD in laying hen model

**Fig. 1:**
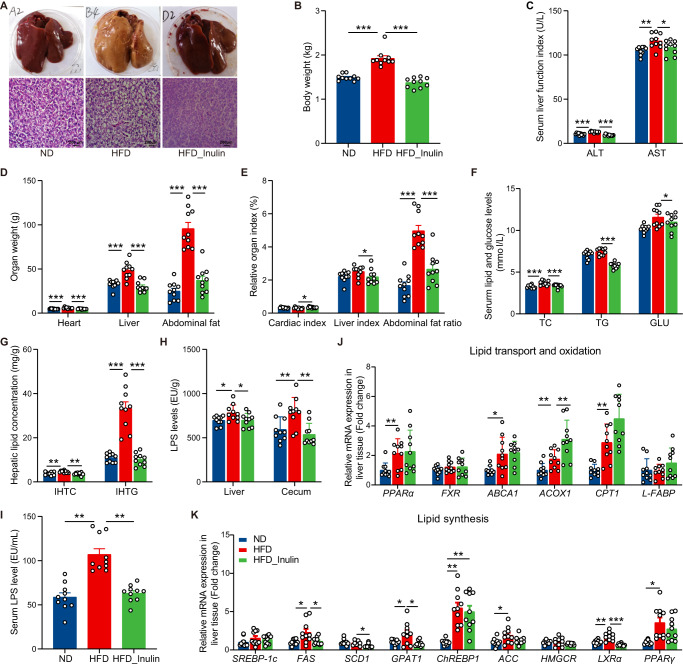
Inulin relieves HFD-induced MAFLD in laying hens. **A** Representative photomicrographs of liver tissues with H&E staining (Scale bars = 200 µm) and phenotype pictures. **B** Body weight (kg). **C** Serum liver function index (U/L). ALT glutamic alanine transaminase, AST glutamic oxaloacetic transaminase. **D** Organ weight (g). **E** Organ index (%, organ weight (g)/body weight (g)*100%). **F** Serum lipid and glucose levels (mmol/L). TC total cholesterol, TG triglyceride, GLU glucose. **G** Hepatic lipid levels (mg/g liver). IHTG intrahepatic triglyceride, IHTC intrahepatic cholesterol. **H** LPS levels of liver tissue and cecum tissue (EU/g). **I** LPS levels of serum (EU/mL). **J** The relative mRNA expression of genes involved in the regulation of lipid transport and oxidation in liver tissues. **K** The relative mRNA expression of genes involved in the regulation of lipid synthesis in liver tissues. Data are presented as the mean ± SEM; *n* = 9–10 hens per group. Statistical analysis was performed using one-way ANOVA followed by the Least Significant Difference (LSD). *P < 0.05, **P < 0.01, ***P < 0.001.

To evaluate the effect of inulin on MAFLD, we treated HFD-induced MAFLD hens by adding 2% inulin to the diet for a period of 16 weeks. Compared to the HFD group, the HFD_Inulin group apparently reduced macrosteatosis and hepatocyte ballooning in the livers of MAFLD hens, as indicated by H&E staining of liver sections (Fig. 1A). The HFD_Inulin group showed a significant reduction in body weight (Fig. 1B), serum liver function index (Fig. 1C), organ weight (Fig. 1D) and organ index (Fig. 1E). Moreover, inulin treatment effectively improved the disorder of lipid and glucose metabolism in MAFLD hens by decreasing serum lipid and glucose levels (Fig. 1F). Compared with the intrahepatic lipid level in the HFD group, the intrahepatic triglyceride (IHTG) and the intrahepatic cholesterol (IHTC) level in the HFD_Inulin group was decreased by 69.54% and 21.04%, respectively (Fig. 1G). MAFLD, which is characterized by an increase of IHTG content that can be accompanied by inflammation^14^. We found that inulin supplementation greatly lowered the levels of LPS in the serum, liver tissue and cecum tissue of MAFLD hens (Fig. 1H, I), thereby alleviating liver injury and inflammation. Additionally, inulin markedly promoted the expression of genes related to lipid oxidation (acyl-Coenzyme A oxidase 1, *ACOX1*) (Fig. 1J), and suppressed the expression of genes related to lipid synthesis, such as fatty acid synthase (*FAS*), stearyl-CoA desaturase 1 (*SCD1*), glycerol-3-phosphate acyltransferase 1 (*GPAT1*) and liver X receptors α (*LXRα*) (Fig. 1K). Overall, dietary inulin effectively reversed the features of MAFLD and metabolic disorders in HFD-fed hens.

### The anti-MAFLD effect of inulin is associated with gut microbiota changes

**Fig. 2:**
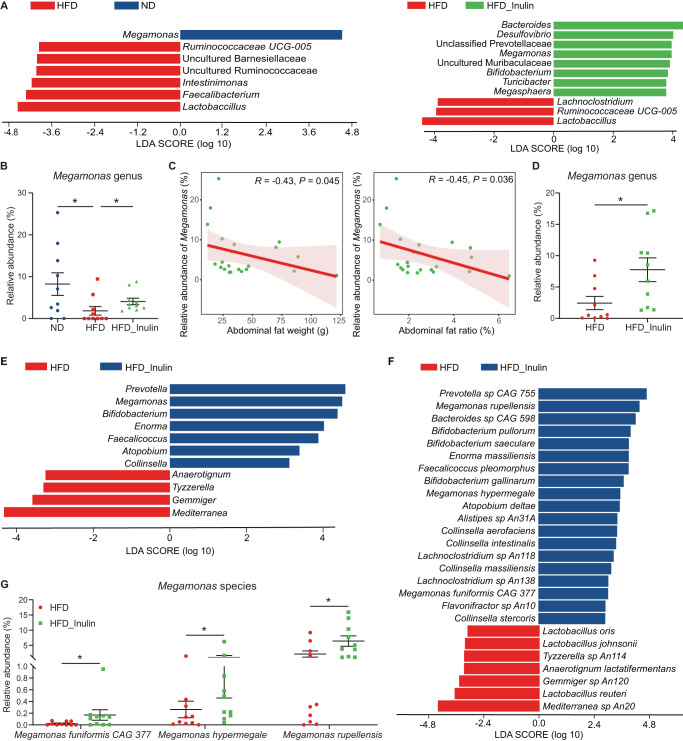
The impacts of inulin on cecal microbiota of HFD-induced MAFLD laying hens. **A**–**C** Cecal microbial analysis based on 16S rRNA gene sequencing data. **A** Differences in the bacterial communities between different groups at the genus level. Significant differences in Linear discriminant analysis (LDA) scores (*P* < 0.05) were produced between groups (Wilcoxon’s test). The threshold of the logarithmic LDA score was 2.0. **B** Relative abundance of *Megamonas*. Data were analyzed using non-parametric Kruskal–Wallis test. **C** Spearman’s correlation analysis between the relative abundance of inulin-enriched *Megamonas* and abdominal fat weight and fat ratio. Lines represent the linear model fit. R, correlation coefficient. Samples with non-detectable levels of *Megamonas* were not included. **D**–**G** Cecal microbial analysis based on metagenomic data. **D** Relative abundance of *Megamonas* genus in cecum samples of hens analyzed by using non-parametric Mann–Whitney U test. LDA scores of differentially abundant genus (**E**) and species (**F**) between the HFD group and HFD_Inulin group using the LEfSe method. **G** Relative abundance of species from *Megamonas* genus in cecum samples of hens analyzed by using non-parametric Mann–Whitney U test. *n* = 10 hens per group, *P < 0.05.

We then profiled the ileal and cecal microbiota to reveal the effect of inulin on microbes in different chicken gut segments. We found that inulin did not alter either the α-diversity (the Shannon index) or β-diversity of ileal microbiota (PCoA) (Supplementary Fig. 1A, B), and no differentially abundant taxa were observed at both the phylum (Supplementary Fig. 1C) and the genus levels (Supplementary Fig. 1D). In contrast, a clear separation of the cecal microbiota among the three groups were found, despite no differences in Shannon diversity index (Supplementary Fig. 2A, B). And 4, 31 and 37 cecal amplicon sequence variants (ASVs) were found unique in the ND, HFD and HFD_Inulin groups, respectively, while 76 (51.35%) ASVs were shared (Supplementary Fig. 2C). Linear discriminant analysis (LDA) effect size (LEfSe) analysis revealed obvious differences in the cecal bacterial communities between different groups. At the genus level, compared with the ND group, HFD significantly reduced the abundance of *Megamonas* but increased the abundance of *Ruminococcaceae UCG-005*, uncultured *Barnesiellaceae*, uncultured *Ruminococcaceae*, *Intestinimonas*, *Faecalibacterium* and *Lactobacillus* (Fig. 2A). After inulin intervention, the abundances of *Lachnoclostridium*, *Ruminococcaceae UCG-005* and *Lactobacillus* were lowered, while *Bacteroides*, *Desulfovibrio*, unclassified *Prevotellaceae, Megamonas*, uncultured *Muribaculaceae*, *Bifidobacterium*, *Turicibacter* and *Megasphaera* were significantly enriched (Fig. 2B).

### *Megamonas* is responsible for the anti-MAFLD effect of inulin

We noticed that *Megamonas* was the only genus that was significantly reduced by HFD but rescued by inulin (Fig. 2A, B), which prompted us to investigate the relationship between this genus and MAFLD. Correlation analysis showed that the abundance of *Megamonas* was significantly negatively correlated with abdominal fat weight and ratio (Fig. 2C). Therefore, it is reasonable to speculate that *Megamonas* may play a key role in alleviating MAFLD. We then use an independent cohort of old laying hens consisting of healthy control (Ctr) and fatty liver (FL) groups to validate this. The hepatocyte injury and ballooning indicated by H&E staining in the FL group was significantly severe than that in the Ctr group (Supplementary Fig. 3A), and hepatic steatosis in the Ctr group was significantly lower than that in the FL group as indicated by lower IHTG and IHTC in the Ctr group (Supplementary Fig. 3B). The FL group also displayed increased expression levels of genes related to lipid synthesis (such as *SREBP-1c*, *FAS*, *SCD1*, *GPAT*, *ChREBP*, *LXRα* and *ACC*) (Supplementary Fig. 3C). Interestingly, cecal microbiota profiling results showed that *Megamonas* was significantly decreased in the FL hens (Supplementary Fig. 4A, B), and the abundance of *Megamonas* was significantly negatively correlated with IHTG and IHTC (Supplementary Fig. 4C). These results strongly suggested the possible role of *Megamonas* in ameliorating MAFLD.

As showed that inulin may promote the enrichment of *Megamonas* bacteria (Fig. 2A, B), we next applied metagenomic sequencing to find the species-level abundance changes within this genus. Consistent with the results of 16S rRNA gene amplicon sequencing, at the genus level, we found that *Megamonas* was significantly enriched in the HFD_inulin group (Fig. 2D–E), and the abundance of *Megamonas* was significantly negatively correlated or tended to be negatively correlated with different MAFLD traits (Supplementary Fig. 5). Among 19 species enriched in the inulin-supplemented hens, three were *Megamonas* species, i.e., *Megamonas rupellensis*, *Megamonas funiformis CAG377* and *Megamonas hypermegale* (Fig. 2F, G).

### Culturomics and identification of *Megamonas funiformis* CML154

To isolate these inulin-enriched *Megamonas* species, culturomics was performed on the cecal samples of HFD_Inulin group hens (Supplementary Fig. 6). Different media including LB, GAM, and FAA were used to isolate and cultivate the cecal microorganisms of laying hens under anaerobic and aerobic conditions (Supplementary Table 1). About 3000 colonies were picked from plates prepared for all cultivation conditions, and a total of 193 species were obtained in pure culture; most of them belonged to the phylum Firmicutes (*n* = 140, 72.54%), followed by members of the phyla Actinobacteria (*n* = 22, 11.40%), Proteobacteria (*n* = 21, 10.88%), Bacteroidetes (*n* = 9, 4.66%) and Fusobacteria (*n* = 1, 0.52%). In detail, *Bacillales*, *Lactobacillales* and *Clostridiales* were the most frequently isolated bacterial orders, representing 39.90%, 18.65% and 9.84% of the total isolates, respectively (Supplementary Fig. 7 and Supplementary Table 2). Among these cultured species, one was identified as *Megamonas funiformis* from the GAM medium, and the corresponding strain was named *M*. *funiformis* CML154. The optimal growth conditions of strain CML154 were 37 °C and anaerobic, and the colonies cultured on GAM agar medium for 48 h are light yellow, flat, with irregular wavy edges, low water content, and about 2-3 mm in diameter (Supplementary Fig. 8A). Additionally, *M*. *funiformis* CML154 was large rod-shaped bacteria observed by light micrographs and scanning electron micrographs (Supplementary Fig. 8B–D).

### *M. funiformis* CML154 prevents diet-induced MAFLD in laying hen model

**Fig. 3:**
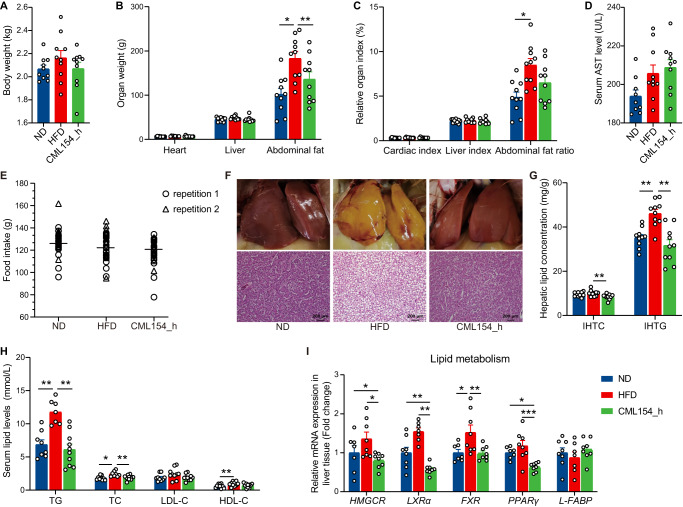
*M. funiformis* CML154 attenuates hepatic steatosis and MAFLD in laying hens. **A** Body weight (kg). **B** Organ weight (g). **C** Organ index (%). **D** Serum liver function index (U/L). **E** Food intake (g). Each symbol represents averaged daily food intake per hen measured every week over a 19-week period (triangles and circles represent two different repetitions). **F** Representative photomicrographs of liver tissues with H&E staining (Scale bars = 200 µm) and Phenotype picture. **G** Hepatic lipid levels (mg/g liver). **H** Serum lipid levels (mmol/L). **I** The relative expression of genes related to lipid metabolism in the liver tissues. Data are presented as the mean ± SEM; *n* = 7–10 hens per group. Statistical analysis was performed using one-way ANOVA followed by the LSD. *P < 0.05, **P < 0.01, ***P < 0.001.

To investigate whether M. funiformis CML154 has anti-MAFLD effect, hens were maintained on either normal chow diet or HFD with or without oral gavage of M. funiformis CML154 for 19 weeks. The results showed that *M. funiformis* CML154 did not affect the birds’ body weight, food intake, heart weight, liver weight, organ index and serum AST (Fig. 3A–E), but significantly decreased the abdominal fat weight (Fig. 3B). Notably, oral *M. funiformis* CML154 significantly attenuated hepatic steatosis (Fig. 3F), reduced the accumulation of hepatic and serum lipids (TG and TC) (Fig. 3G, H), and decreased the expression of lipid metabolism related genes such as *HMGCR*, *LXRα*, *FXR* and *PPARγ* (Fig. 3I). Moreover, 16S rRNA microbial profiling results showed that still there was a negative correlation between the relative abundance of *Megamonas* and intrahepatic lipid content (Supplementary Fig. 9). These results confirmed the role of *M. funiformis* CML154 in attenuating HFD-induced MAFLD in laying hens.

### *M*. *funiformis* CML154 prevents diet-induced MAFLD in mouse model

**Fig. 4:**
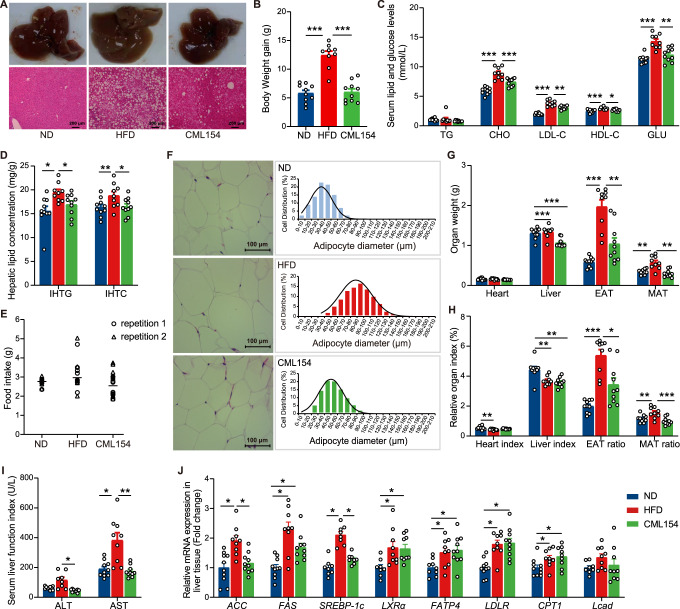
*M*. *funiformis* CML154 attenuates hepatic steatosis and MAFLD in mice. **A** Representative photomicrographs of liver tissues with H&E staining (Scale bars = 200 µm) and phenotype picture. **B** Body weight gain (g). **C** Serum lipid and glucose levels (mmol/L). **D** Hepatic lipid levels (mg/g liver). **E** Food intake (g). Each symbol represents averaged daily food intake per mouse measured every week over a nine-week period (triangles and circles represent two different cages). **F** Representative H&E-stained pictures (Scale bars = 100 µm) and adipocytes diameter (µm) distribution of EAT. Scale bar = 100 µm. **G** Organ weight (g). **H** Organ index (%). **I** Serum liver function index (U/L). **J** Relative expression of genes related to lipid metabolism in the liver tissues. Data are presented as the mean ± SEM. *n* = 8–10 mice per group. Statistical analysis was performed using one-way ANOVA followed by the LSD. *P < 0.05, **P < 0.01, ***P < 0.001.

We then explored whether the effect of *M. funiformis* CML154 in alleviating MAFLD is host species-specific. Given mice are the most commonly used animal model for studying fatty liver disease, male C57BL/6J mice were maintained on either ND or HFD with or without oral gavage of *M. funiformis* CML154 for 9 weeks. The results showed that *M. funiformis* CML154 significantly attenuated hepatic steatosis (Fig. 4A) and body weight gain (Fig. 4B), as well as decreased serum glycolipid level (Fig. 4C) and intrahepatic lipid level (Fig. 4D), while had no significant effect on mice food intake (Fig. 4E). It is reported that obesity and associated metabolic diseases is associated with adipocyte hypertrophy, which contributes to ectopic fat deposition^15^. We found that *M*. *funiformis* CML154 treatment significantly decreased the proportion of large adipocytes (diameter ≥ 80 µm), increased the proportion of small adipocytes (diameter ≤ 60 µm) in the epididymal adipose tissue (EAT) (Fig. 4F), and reduced EAT weight, mesenteric adipose tissue (MAT) weight, EAT ratio and MAT ratio (Fig. 4G, H). In addition, *M*. *funiformis* CML154 treated mice had lower liver weight, liver index and the levels of serum ALT and AST (Fig. 4G–I). Moreover, oral *M. funiformis* CML154 significantly inhibited the expression of *ACC* and *SREBP-1c* (Fig. 4J).

### *M*. *funiformis* CML154 regulates hepatic gene expression profiles in mice

**Fig. 5:**
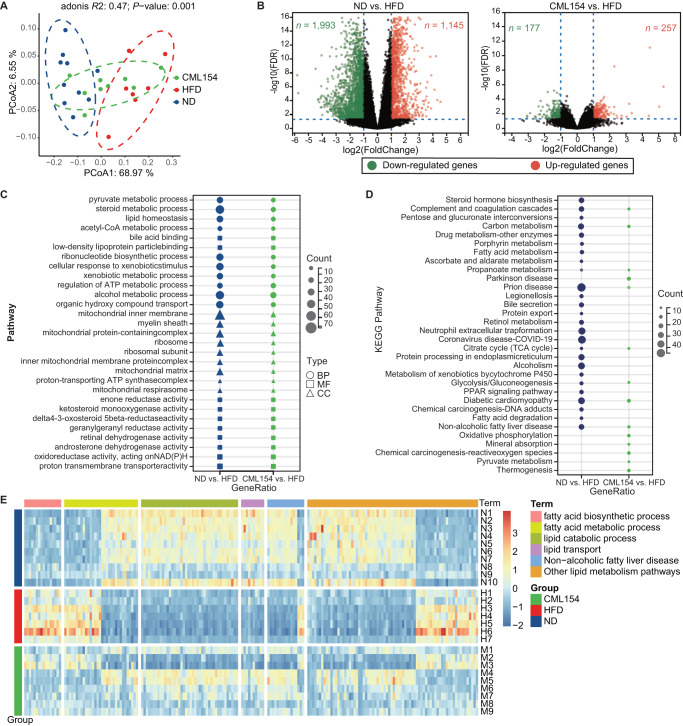
*M*. *funiformis* CML154 regulates hepatic gene expression in HFD-fed mice. **A** PCoA analysis. **B** Volcano plot of DGEs. **C** GO enrichment analysis. BP Biological Process, MF Molecular Function, CC Cellular Component. **D** KEGG pathway analysis. **E** Heatmap cluster analysis of 190 DGEs related to lipid metabolism. Adjusted *P* values for RNA-seq were obtained using the Wald test, including adjustment for multiple testing (Benjamini–Hochberg). *n* = 7–10 mice per group.

To understand the potential mechanisms underlying the anti-MAFLD effect of *M. funiformis* CML154, the liver gene expression profiles were investigated by transcriptomic analysis in control and HFD mice with or without CML154 treatment. PCoA showed that the gene expression profiles were very different among ND, HFD, and CML154 groups (Fig. 5A). Mice-induced by HFD displayed 3138 differentially expressed genes (DEGs) compared with the ND mice, and CML154 treatment resulted in 434 DEGs compared with the HFD group, among which 112 were upregulated and 322 were downregulated (Fig. 5B). Compared with their respective controls, DEGs in the HFD group were enriched in 556 GO terms (Supplementary Table 3), and DEGs in the HFD_CML154 group were enriched in 127 GO terms (Supplementary Table 4). Moreover, a total of 322 DGEs in the two groups were commonly enriched in 73 GO terms mainly belonging to Biological Process (BP), Molecular Function (MF), and Cellular Component (CC). Among them, five GO terms including pyruvate metabolic process (GO:0006090), steroid metabolic process (GO:0008202), lipid homeostasis (GO:0055088), bile acid binding (GO:0032052), and low-density lipoprotein particle binding (GO:0030169) were closely related to lipid metabolism (Fig. 5C). KEGG analysis showed that DGEs in the ND and CML154 groups were enriched in 27 and 14 metabolic pathways (Supplementary Tables 5 and 6), respectively, among which Propanoate metabolism and Non-alcoholic fatty liver disease pathways were commonly affected (Fig. 5D). Further, heatmap cluster analysis of 190 DEGs related to lipid metabolism revealed that, compared with the ND mice, genes related to fatty acid biosynthetic process were significantly up-regulated in the HFD mice, while were suppressed by CML154 treatment; in contrast, genes related to lipid catabolic process and lipid transport were significantly down-regulated in HFD mice but up-regulated in CML154 treated mice (Fig. 5E). These results suggested that the anti-MAFLD effect of CML154 was associated with the inhibition of fatty acid de novo synthesis and promotion of β-oxidation in liver of HFD-fed mice.

### The anti-MAFLD effect of *M*. *funiformis* CML154 is associated with its production of propionic acid

**Fig. 6:**
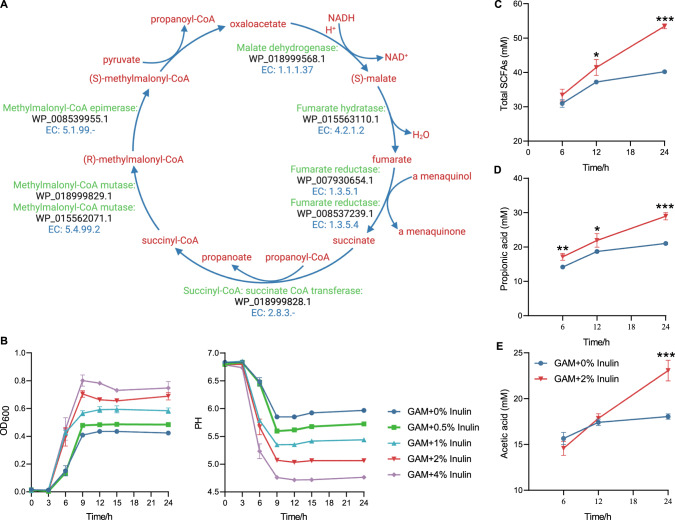
Inulin promotes the growth of *M*. *funiformis* CML154 and its production of propionic acid. **A** Propionate metabolism pathway in *M*. *funiformis* CML154. **B** Growth curve and pH value changes in fermentation broth of *M*. *funiformis* CML154 with different concentrations of inulin added. The production of total SCFAs (**C**), propionic acid (**D**), and acetic acid (**E**) during *M*. *funiformis* CML154 growth with or without 2% inulin added. *n* = 4 per treatment group. Data are presented as the mean ± SEM. Statistical analysis was performed using independent-samples *t* test. *P < 0.05, **P < 0.01, ***P < 0.001.

To further explore why *M*. *funiformis* CML154 attenuates MAFLD, we sequenced and analyzed its whole genome (Supplementary Fig. 10A; Supplementary Table 7). The genome of *M*. *funiformis* CML154 displayed 97.7% average nucleotide identity (ANI) to the *M*. *funiformis* type strain YIT 11815. Phylogenetic analysis based on whole genome SNPs showed that the *M*. *funiformis* CML154 was most related to *M. funiformis* YIT 11815 and *M*. *funiformis* JCM14723 of human origin (Supplementary Fig. 10B). Genome-based metabolic modeling results indicated that *M*. *funiformis* CML154 can metabolize fructose, fucose, arabinose, trehalose, stachyose, etc., and can synthesize folate and biotin (Supplementary Table 8). A total of 1833 genes from the genome were annotated in 20 KEGG pathways belonging to five major functions including metabolism, genetic information processing, environmental information processing, cellular process and human disease (Supplementary Fig. 11A). At the sub-metabolic pathway classification level, CML154 was found involved in various processes related to glycolipid and energy metabolism (Supplementary Fig. 11B–E, Supplementary Table 9). Notably, in the carbohydrate metabolism category, 13 genes were found associated with propanoate metabolism (Supplementary Fig. 11E), suggesting that CML154 was a propionic acid-producing bacterium, which was further confirmed by the fact that nearly all enzymes involved in the pyruvate fermentation to propanoate I pathway were encoded by the CML154 genome (Fig. 6A). In vitro cultivation results verified that, after 24 h fermentation using GAM medium under anaerobic condition, propionate was the major SCFA produced by CML154 (21 mM), followed by acetate (18 mM), which was in consistent with previous findings^16^.

We then tested if inulin can promote the growth of *M*. *funiformis* CML154 and stimulate its production of SCFAs in vitro. The results showed that inulin clearly promoted the growth of CML154 and decreased the medium pH values in a dose-dependent manner (Fig. 6B). Taken 2% inulin addition as an example, the production of propionic acid at all three time points (6 h, 12 h, and 24 h) measured were significantly higher than that in the control medium, while the production of acetic acid was only increased at 24 h (Fig. 6C–E). This may suggest that along with the growth of *M*. *funiformis* CML154, propionic acid was consistently produced, while acetic acid was just generated at the stationary phase. We therefore hypothesized that the effects of *M*. *funiformis* on relieving MAFLD in laying hen and mouse models were probably due to the propionic acid it produced. This can be supported by all our in vivo studies above: inulin supplementation significantly increased the levels of propionate in cecum compared with the HFD-induced MAFLD hens (Supplementary Fig. 12A); in the validation cohort of aged hens, the cecal concentration of propionate was significantly higher in healthy hens than that in MAFLD hens (Supplementary Fig. 12B); and after *M*. *funiformis* CML154 administration, the levels of total SCFAs, propionate, and acetate in cecum of both hens and mice were increased (Supplementary Fig. 12C, D).

### Propionate reduces lipid in hepatocytes by activating the *APN*-*AMPK*-*PPARα* signal pathway

**Fig. 7:**
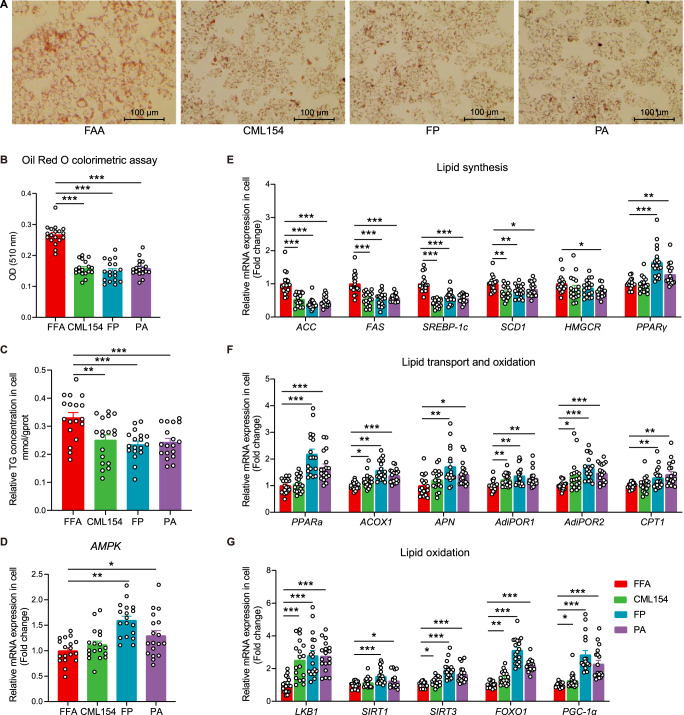
Propionic acid reduced lipid in hepatocytes by activating the *AMPK* signal pathway. HepG2 cells induced with 0.5 mM FFA (oleic acid: palmitic acid = 2:1) for 24 h were treated with live *M*. *funiformis* CML154 (CML154), its fermentation products in FBS- and antibiotic-free DMEM (FP), and propionate (PA) for 6 h, and then cells were harvested for the indicated analysis. **A** Representative photomicrographs of hepatocytes with Oil red O staining (Scale bars = 100 µm). **B** Oil red O colorimetric assay. **C** TG level in hepatocytes. **D**–**G** The relative mRNA expression in hepatocytes. Data are presented as the mean ± SEM; *n* = 6 wells per group, and each experiment was repeated three times. Statistical analysis was performed using one-way ANOVA followed by the LSD. *P < 0.05, **P < 0.01, ***P < 0.001.

To validate if the anti-MAFLD activity of *M*. *funiformis* CML154 is mediated by propionate it produced and to reveal the underlying mode of action, we constructed hepatic steatosis model using hepatocytes (HePG2 cell), which was then treated with live *M*. *funiformis* CML154 (CML154), its fermentation products in cell culture medium DMEM (FP), and propionate (PA). Through a series of preliminary experiments, we determined that the optimal concentration of FFA to induce hepatocyte steatosis was 0.5 mM (Supplementary Fig. 13A–C); the optimal concentration and time of live CML154 to stimulate cells were 1 × 10^7^ CFU/mL and 6 h (Supplementary Fig. 13D–G), respectively; the concentration of propionate added was according to its content in CML154 fermentation supernatant in DMEM medium (Supplementary Fig. 13H). The results showed that the addition of live CML154, its fermentation products or propionate efficiently decreased the cellular lipid content (Fig. 7A–C). This reduction occurred with decreased expression levels of genes related to lipid synthesis, such as *ACC*, *FAS*, *SREBP-1c*, *SCD1* and *PPARγ*, and increased expression levels of *AMPK* and lipid transport and oxidation-related genes (Fig. 7D–G). As studies have shown that propionate can activate *AMPK*^17^, we thus focused on the related pathway and found that the upstream (*APN*, *AdiPOR1*, *AdiPOR2* and *LKB1*) and downstream (*SIRT1*, *SIRT3*, *FOXO1*, *PGC-1α* and *ACOX1*) genes of *AMPK* pathway were significantly up-regulated, indicating that the *APN*-*AMPK*-*PPARα* pathway was activated by these treatments. We then checked the *AMPK* expression in the *M*. *funiformis* CML154-gavaged MAFLD laying hens and mice and found that the expression of *AMPK* was significantly increased in the liver tissue of these animals compared with their respective controls (Supplementary Fig. 14). These data confirmed that *M*. *funiformis* CML154 inhibited the lipid synthesis and promoted lipid transport and oxidation and activated the *APN*-*AMPK*-*PPARα* pathway by producing propionate, and thereby reduced lipid deposition in hepatocytes.

## Discussion

In this study, we constructed a laying hen model of MAFLD by dietary HFD intervention, and demonstrated for the first time that the inulin-enriched *Megamonas* had a cross-species effect in ameliorating MAFLD. Although diet-induced or genetically modified mice or rat are most commonly used MAFLD models, other animal species such as zebrafish, fruit flies, rabbit, Ossabaw pig, and chickens have also been studied for revealing the MAFLD pathogenesis and therapeutic targets^18,19^. As for chickens, it is suggested that they are more predisposed to fat deposition in the liver, which resembles the hallmark of MAFLD in human clinics that marked accumulation of triglycerides within hepatocytes. In our laying hen model, the highly expressed genes, *FAS* and *CPT1*, related to lipid synthesis and oxidation, respectively, were also found highly expressed in our following diet-induced mice model, suggesting that common targets were regulated by the inulin-enriched *Megamonas* in the two animal species.

Accumulated evidences have shown that manipulating gut microbiota using prebiotics and probiotics may be a viable therapeutic strategy for improving MAFLD^3,4,6,20^. Inulin is a well-studied prebiotic that can modulate the gut microbiota and thus exert a lipid-lowing effect^7^. This is in accordance with our results that inulin intervention significantly reduced the MAFLD hens’ serum and hepatic TC and TG contents. Although studies have suggested that inulin was selectively fermented by human and animal gut *Bifidobacteria* (we also observed in this study), we found that *Megamonas* was subjected to the most dramatic changes before and after inulin intervention in the MAFLD laying hens. Our results further showed that inulin significantly stimulated the growth of *Megamonas* isolate *M. funiformis* CML154 in a dose-dependent manner, for the first time, providing direct evidence that inulin has the ability to promote the growth of *Megamonas* bacteria.

Currently, the role of *Megamonas* in human health is unclear. The abundance of this genus was significantly decreased in gut microbiota of patients with type 1 diabetes^21^, heart failure^22^, multiple system atrophy^23^, spinal cord injury^24^, and Behcet’s disease^25^. Moreover, *Megamonas* was demonstrated to be a core taxon in healthy Japanese populations^26^, and the abundance of *Megamonas* was also found increased in children with higher physical activity^27^. All these facts indicate that *Megamonas* may have beneficial effects on human health. As for the role of *Megamonas* in obesity and metabolic diseases, conflicting results were reported. Studies found that *Megamonas* was over-represented in overweight and obese people^28^, and was associated with MAFLD and NASH severity^29,30^, however, the following verification experiment failed to establish a causal relationship between an *M. funiformis* isolate and MAFLD in mice^30^. In contrast, a recent study showed that the abundance of *Megamonas* was significantly decreased in high animal fat diet human individuals^31^. Our results revealed that oral gavage of *M. funiformis* CML154 markedly alleviated hepatic steatosis in both laying hen and mouse MAFLD models, providing a causal proof of the negative associations between *Megamonas* and hepatic steatosis-related parameters we observed and demonstrated that species in the genus *Megamonas* paly a beneficial role in the regulation of host lipid metabolism.

The species *M. funiformis* was first identified from healthy Japanese males in 2008, and was recognized as a SCFA-producer, mainly propionic acid^32^. Propionate has been identified as the principal hepatic gluconeogenic substrate and was found having multiple metabolic benefits, including improving insulin sensitivity, glucose and lipid metabolism^33^. Recent studies showed that long-term oral propionate prevents weight gain and reduces lipid accumulation in obese humans^34,35^. Also, in vitro isotope labeling experiments confirmed the effect of propionate in inhibiting hepatocyte cholesterol and fatty acid biosynthesis^36^, which was in consistent with the observations in our hepatic steatosis model. Propionate has been proved to activate the phosphorylation of *AMPK* in HCT116, 3T3-L1 and HepG2 cells in vitro^37,38^*. AMPK* is a key energy and metabolic regulator that senses energy status and regulates energy consumption and storage^39^, which plays an important role in the occurrence and development of MAFLD^40^. The activation of *AMPK* and associated pathways inhibits the occurrence and development of MAFLD mainly through three mechanisms: inhibiting hepatic lipogenesis, enhancing fatty acid oxidation, and improving hepatic mitochondrial function^40^. In our mice model, liver transcriptomic data clearly showed that administration of *M. funiformis* CML154 significantly suppressed pathways related to fatty acid biosynthetic process but up-regulated genes related to lipid catabolic process. The validation of the propionate-mediated anti-MAFLD activity of *M. funiformis* CML154 in hepatic steatosis cell model further confirmed this mode of action, and verified the activation of *APN*-*AMPK*-*PPARα* pathway by propionate. These previous findings coupled with our results suggest that propionic acid produced by the gut microbes, *M. funiformis* CML154 for example, plays a critical role in attenuating obesity and fatty liver diseases.

**Fig. 8:**
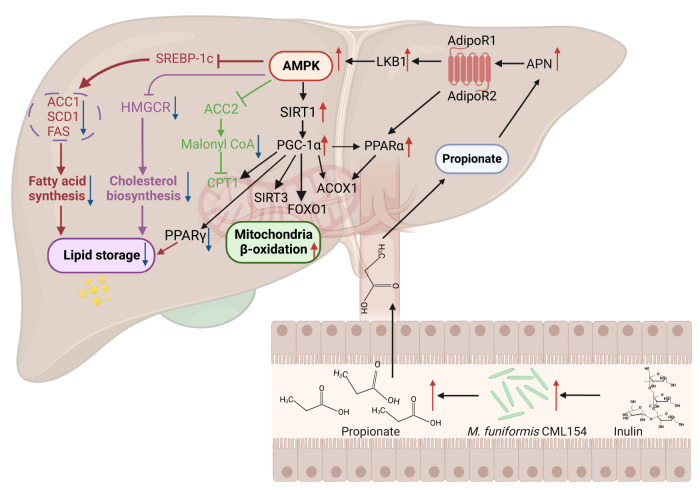
Proposed mechanisms underlying the anti-MAFLD effect of inulin mediated by *M. funiformis* CML154. Dietary inulin modulates the gut microbiota by promoting the growth of *M. funiformis* CML154. Propionic acid produced by *M. funiformis* CML154 enters liver through the hepatic portal vein, where it activates *APN*, which in turn is responsible for activating *AMPK* and *PPARα* pathways via binding to its receptors *AdipoR1* and *AdipoR2*. The activation of *PPARα* together with *ACOX1* participates in regulating lipid oxidation. Simultaneously, activated *AMPK* inhibits hepatic lipogenesis, enhances fatty acid oxidation, and improves hepatic mitochondrial function through regulating downstream target genes.

In conclusion, we provide a new clue that the MAFLD-alleviating effect of inulin is associated with the genus *Megamonas* in gut microbiota. The inulin-enriched isolate *M*. *funiformis* CML154 displayed a cross-species (in both laying hens and mice) effect in ameliorating MAFLD. The lipid-lowing activity of *M*. *funiformis* CML154 was attributed to the activation of the *AMPK* pathways by its major fermentation end product, propionic acid (Fig. 8). Collectively, our findings evidenced new interactions in the gut-liver axis by connecting inulin, *Megamonas*, propionate, and MAFLD, and the newly isolated intestinal strain *M*. *funiformis* CML154 may be a potential probiotic to prevent and treat MAFLD and other metabolic disorders. We should stress that, as the three species of *Megamonas* were all found highly represented in the inulin-treated MAFLD laying hen models, a comparison of the roles of strains from *M*. *funiformis*, *M. hypermegale* and *M. rupellensis* in alleviating MAFLD is worthy of further investigation.

## Methods

### Ethics approval

All animal experimental procedures were conducted according to guidelines of the Institutional Studies Animal Care and Use Committee of China Agricultural University (Beijing, China). The study was approved by the Ethics Committees of Laboratory Animal Center of China Agricultural University (Beijing, China) (permit AW13501202-2-2).

### Chicken raising

HY-Line gray commercial laying hens were purchased and housed in Zhuozhou breeding base of China Agricultural University (Zhuozhou, China). All hens were housed in pairs in stainless-steel ladder cages (45 × 45 × 45 cm) and in a controlled room, in which temperature and relative humidity was maintained at 26 ± 2 °C and 50 ± 10%, respectively. The photoperiod was a 16 h light/8 h dark with a dark cycle encompassing 10 pm to 6 am. All the birds were allowed free access to water and food. During the experiments, food intake was recorded once a week. All the birds were subjected to an acclimatization period of one week before formal test, during which they were fed a normal diet (ND), and met the NRC^41^ and the management manual of Hy-Line gray laying hens’ recommendations. The ingredients and nutrient levels of ND are presented in Supplementary Table 10.

### HFD induction and inulin intervention

A total of 45 24-week-old Hy-Line gray commercial laying hens were divided into three groups based on their body weight, with three replicates per group and 15 hens per replicate. Laying hens were fed for 16 weeks with ND, or High-Energy-low-Protein Diet (HFD) with or without inulin supplementation (2% in HFD). The composition and nutrient levels of HFD are shown in Supplementary Table 10.

### Validation of microbiota changes using an old laying hen cohort

We randomly selected 75 85-week-old hens from a normal aging Hy-Line brown commercial laying hen population. These hens were euthanized, and liver tissue were collected and fixed with 4% paraformaldehyde to make liver pathological tissue sections, based on which we assessed degree of hepatic steatosis for these hens and divided them into healthy control group (Ctr, *n* = 13) and fatty liver group (FL, *n* = 62). Then, we randomly selected 8 hens from each of these two groups for subsequent analyses.

### Culturomics

The collected cecal content of HFD_Inulin group hens were immediately aliquoted and send to CAST MicroLab (China Agricultural University, Beijing, China) on dry ice, and frozen at −80 °C for less than 3 months before use. The cecal contents were opened and processed under anaerobic conditions in a Whitley DG250 workstation (10% H_2_, 10% CO_2_ and 80% N_2_ atmosphere; Don Whitley Scientific DG250, West Yorkshire, UK) at 37 °C. Pre-reduced phosphate buffered saline (PBS) supplemented with 0.1% cysteine, agar plates with different growth media (Supplementary Table 1), and other materials that were used for culturing were placed in the anaerobic incubator overnight before use to reduce to anaerobic conditions. The samples were divided in two. One part was directly homogenized in pre-reduced PBS (1 g of content was squeezed into 9 mL pre-reduced PBS^42^) and was serially diluted and spread directly on 12 different agar plates (Supplementary Table 1). The other part was pre-incubation in various liquid culture media under anaerobic conditions, and then diluted with pre-reduced PBS and plated onto agar plates with different growth media. All plates were incubated at 37 °C for 2–7 d. Well-separated colonies of different morphology were picked from each agar plate and streaked onto new plates to obtain single clones. Each isolate was identified by PCR amplification of the 16S rRNA gene followed by sequencing, and amplification primers were 27F (5′-AGAGTTTGATCCTGGCTCAG-3′) and 1492R (5′-ACGGCTACCTTGTTACGACTT-3′). The sequences were compared with the NCBI database or EzBioCloud (http://www.ezbiocloud.net)^43^ for taxonomic affiliation. Then, all identified isolates were stored in the corresponding growth medium containing glycerol at 20% concentration at −80 °C (Supplementary Fig. 8).

### Assessment the impact of inulin on the growth of *M. funiformis* CML154

*M. funiformis* CML154 was inoculated in modified Gifu Anaerobic Medium (GAM) broth (HB8518-3, hopebio, Qingdao, China) supplemented with different doses of inulin (0%, 0.5%, 1%, 2% and 4%) and incubated for 24 h under anaerobic condition. Samples were collected at the 0, 3^rd^, 6^th^, 9^th^, 12^th^, 15^th^ and 24^th^ h, and then the absorbance at 600 nm was measured using a Microplate Reader (BioTeK Epoch2, American Berten Instrument Co., Ltd, USA) and the pH was detected using a pH meter (FiveEasy Plus, METTLER TOLEDO, Shanghai, China). Among which, the samples supplemented with or without 2% inulin were measured SCFAs with targeted metabolomics at 6^th^, 12^th^ and 24^th^ h. Of note, there were three replicates per treatment group.

### Preparation of *M. funiformis* CML154 suspension for oral gavage

*M. funiformis* CML154 was grown at 37 °C with GAM for 24 h, and the bacterial cells were obtained by centrifugation at 4000 rpm for 5 min at 4 °C, resuspended in sterile anaerobic PBS with 20% glycerol, and then stored at −80 °C until use. The bacterial suspension was activated in a 37°C water bath for 5 min, and diluted to 1 × 10^8^ CFU/mL (the final concentration of glycerol is 0.8%) with sterile PBS before gavage. In hen experiment, 60 27-week-old Hy-Line gray commercial laying hens were divided into three groups based on their body weight, with 10 replicates per group and two hens per replicate. The control group continued receiving ND while the other two groups were fed HFD throughout the experiment to induce MAFLD. In two MAFLD groups, one group (CML154_h) was treated daily with 5 mL live *M. funiformis* CML154 frozen in anaerobic PBS (1 × 10^8^ CFU/mL), and the other group (HFD) was given an equivalent volume of sterile anaerobic PBS with 0.8% glycerol by oral gavage. Treatments were continued for 19 weeks. In mice experiment, 30 6-week-old C57BL/6J male mice were purchased (Charles river, Beijing, China) and housed in SPF (specific pathogen free) conditions in Auxiliary building of teaching center of College of Biology (China Agricultural University, Beijing, China). Mice were housed in stainless steel cages (five mice/cage) with free access to sterile water and sterile food (irradiated), and kept in a controlled environment (room temperature maintained at 25 ± 1 °C, 12 h dark-light cycle with a dark cycle encompassing 8 p.m. to 8 a.m.). All mice underwent a one-week acclimatization period before formal test, during which they were fed a control diet (ND) (D12450J, Research Diet, New Brunswick, NJ 08901 USA), and food and weight were recorded once a week during the experiments. 30 mice were divided into three groups (*n* = 10) based on body weight and fed with ND or 60% high-fat diet (HFD) (D12492, Research Diet, New Brunswick, NJ 08901 USA). One group (CML154) of HFD-fed mice was treated with live *M. funiformis* CML154 frozen in anaerobic PBS, each mouse was treated with oral administration 0.2 mL of *M. funiformis* CML154 at 1 × 10^8^ CFU/mL per day. ND and HFD groups were given an equivalent volume of sterile anaerobic PBS with 0.8% glycerol by oral gavage. Treatments were continued for 9 weeks. Individual body weights were measured weekly. Averaged daily food intake was measured once a week over a nine-week period for two cages per treatment group (5 mice per cage), and the daily feed intake of each mouse in each cage was calculated according to the calculation formula: average daily feed intake = total feed intake/7d/5 mice^44^.

### Collection of tissue samples

At the end of each animal experiment, animals were fasted for 8 h and were sacrificed under anesthesia by intraperitoneal injection of Zoletil^®^ 50 (Lab Anim Technology, China). Then, blood was collected from the orbital venous plexus of mice, while hen blood was collected from the inferior pterygeal vein. Whole blood was placed in tubes without anticoagulant for 1 h at room temperature, then centrifuged at 3000 rpm for 10 min, and supernatant (serum) were collected, and stored at –20 °C for subsequent test. Then hen and mice were immediately euthanized by cutting off the carotid artery and cervical dislocation, respectively. Tissue samples (liver, heart, abdominal fat tissue, epididymal adipose tissue, mesenteric adipose tissue, ileal content and caecal content) were cut to the appropriate size, immediately immersed in liquid nitrogen, and stored at –80 °C for further analysis. A part of liver and epididymal adipose tissue were fixed in 4% paraformaldehyde for histological analysis.

### Cell experiments

#### Cell Cultures

Human hepatocellular cell (HepG2) was obtained from the Institute of Apicultural Research, CCAS, and cultured in DMEM medium (high glucose) (Gibco, Beijing, China) supplemented with 10% fetal bovine serum (FBS) (NEWZERUM, Christchurch, New Zealand). HepG2 cells were maintained at 37 °C in humidified incubator with 5% CO_2_, and the culture medium was changed every two days.

#### Cell viability assay

The cell viability was evaluated by CCK-8 Cell Proliferation and Cytotoxicity Assay Kit (CA1210, Solarbio, Beijing, China). HepG2 cells were seeded in 96-well culture plates (5 × 10^4^ cells per well) and were maintained at 37 °C in a humidified atmosphere of 5% CO_2_ until to 80% confluence. Cells were then treated according to the purpose of experiment, with six replicates for each treatment. After treatment, the cells were washed with PBS. 10 μL CCK-8 and 100 μL DMEM were then added to each well and incubated for 2 h at 37 °C. The absorbance of each well at 450 nm was read on a Microplate Reader.

#### Oil red O staining

The cells in the different treatment groups were fixed with 4% paraformaldehyde for 30 min at room temperature and stained with Modified Oil Red O Staining Kit (C0158s, Beyotime, Shanghai, China) followed by the manufacturers’ protocols. Photographs were taken under a light microscope (EVOS^TM^ XL Core, Thermo Fisher Scientific, USA). Isopropanol was added to each sample to quantify the Oil red O content, which was shaken at room temperature for 10 min, the absorbance of each 200 μL isopropanol-extracted sample was measured with a Microplate Reader at 510 nm^45^.

#### Examination of cell triglycerides

After treatment, the cells in each well were washed with PBS. 200 μL RIPA (P0013D, Beyotime, Shanghai, China) containing 1% PMSF (ST506-2, Beyotime, Shanghai, China) were then added to each well and lysed on ice for 30 min. The lysed cells were collected into centrifuge tubes and centrifuged at 13,000 rpm for 5 min. The TG content in the collected supernatant was determined by Enhanced BCA Protein Assay Kit (P0009, Beyotime, Shanghai, China) and Triglyceride assay kit (A110-1-1, Jiancheng Bio, Nanjing, China).

#### Establishment of steatosis model in vitro HepG2 cells

HepG2 cells were plated into 6-well cell culture plate (Costar, Corning, NY, USA) by 5 × 10^5^ cells/well and grown until they reach a confluence of more than 80%. HepG2 cells were then exposed for 24 h to a mixture (FFA) of palmitic acid (PA; P0500-10G, Sigma, USA) and oleic acid (OA; O1008-5G, Sigma, USA) at 1:2 molar ratio to a final concentration of 0 mM, 0.5 mM, 1 mM or 1.5 mM^46^. After treatment, the optimal concentration of FFA to induce steatosis in HepG2 cells was determined to be 0.5 mM by measuring cell viability, intracellular TG and Oil Red O staining. The optimal concentration of FFA was used for subsequent cell trials. The experiments were conducted three times with six replicates per treatment group.

#### Determination of the conditions for cell treatment with *M. funiformis* CML154

After *M. funiformis* CML154 was cultured anaerobically at 37 °C for 24 h, the live CML154 was washed twice with PBS at 4000 rpm for 5 min and resuspended in FBS- and antibiotic-free DMEM at the final density of 1 × 10^9^ CFU/mL^45^. Then, CML154 was diluted with FBS- and antibiotic-free DMEM to 1 × 10^8^ CFU/mL, 1 × 10^7^ CFU/mL and 1 × 10^6^ CFU/mL. HepG2 cells were seeded in the bottom of 6-well transwell chambers (Costar, Corning, NY, USA) at 5 × 10^5^ cells per well and were maintained at 37 °C in a humidified atmosphere of 5% CO_2_ until 80% confluence. Then, the confluent cells were washed and cultured in FBS- and antibiotic-free DMEM medium containing 0.5 mM FFA to induce hepatocyte steatosis, and the control group was cultured in FBS- and antibiotic-free DMEM medium. After 24 h induction, HepG2 cells were washed with PBS and treated with 1 mL of DMEM or *M. funiformis* CML154 suspensions at designated concentration (1 × 10^6^, 1 × 10^7^, 1 × 10^8^ and 1 × 10^9^ CFU/mL), which was added into transwell insert (upper chamber), respectively. After 6 h incubation, the cells were washed twice with PBS and tested for cell viability. To determine the time to treat steatosis cells with *M. funiformis* CML154, the cells (5 × 10^5^ cells/well) were seeded into culture plates and grown to 80% confluence, after which 0.5 mM FFA was added to the culture system except the control group, which was incubated for a further 24 h. Then, cells were stimulated with *M. funiformis* CML154 suspensions containing 1 × 10^7^ CFU/mL for 6 h or 24 h at 37 °C. After stimulation, the cells were washed twice with PBS and tested for cell viability. The experiments were conducted three times with six replicates per treatment group.

#### Determination of the conditions for cell treatment with *M. funiformis* CML154 fermentation broth and propionate

*M. funiformis* CML154 was cultured in GAM medium overnight, and washed twice with PBS at 4000 rpm for 5 min and resuspended in FBS- and antibiotic-free DMEM at the final density of 1 × 10^7^ CFU/mL. After 6 h incubation in an anaerobic incubator, bacterial supernatant of *M. funiformis* CML154 were obtained by centrifugation at 4000 rpm for 5 min at 4 °C and passed through a 0.22 μm filter. The SCFAs concentrations in the fermentation broth were detected by an Agilent 5975 C gas chromatograph–mass spectrometer (GC-MS) (Santa Clara, CA, USA). The concentration of propionate in the fermentation broth was determined to be 4 mM. Then, the steatosis cells were treated with DMEM, the fermentation broth, and 4 mM propionate to determine cell viability, and we found that all these treatments had little effect on cell viability The experiments were conducted three times with six replicates per treatment group.

#### Treatment of HepG2 cells with live *M. funiformis* CML154, its fermentation broth, and propionate

HepG2 cells (5 × 10^5^ cells/well) were seeded into culture plates and grown to 80% confluence, after which 0.5 mM FFA was added to the culture system, which was incubated for a further 24 h. Then, cells were stimulated with 1 mL of DMEM, CML154 suspensions (1 × 10^7^ CFU/mL), the fermentation broth, and propionate (4 mM) for 6 h at 37 °C. After treatment, the cells were harvested for subsequent TG and RNA extraction or stained with Modified Oil Red O Staining Kit. The experiments were conducted three times with six replicates per treatment group.

### Serum biochemistry analysis

Levels of serum glucose, triglycerides (TG), total cholesterol (TC, T-CHO), high density lipoprotein cholesterol (HDL-C), low density lipoprotein cholesterol (LDL-C), alanine transaminase (ALT) and aspartate transaminase (AST) were measured by the automatic biochemical analyzer (Kehua ZY KHB-1280, Beijing, China) as instructed by the manufacturer. LPS levels were measured in the serum, liver, and cecum using ELISA Kit (mlbio, Shanghai, China).

### Hepatic lipids examination

Hepatic lipids were extracted according to Folch method^47^ with slight modifications. Briefly, 50 mg of liver tissue was weighted and homogenized in 1 ml of chloroform: methanol (2:1) before agitation at 50 Hz for 30 s with refrigerated high-throughput tissue grinder (SCIENTZ, Shanghai, China). A 200 µL of distilled water was added and vortex to mix, following by centrifugation at 4000 rpm for 10 min and the lower solvent layer was extracted and then dried with a centrifugal vacuum concentrator (Jiaimu CV200, Beijing, China). The residue was resuspended with an equivalent volume of PBS supplemented with 5% Triton X-100 as a total lipid extract sample. The contents of TG and TC in the livers were assayed with the TG assay kits (BIOSINO, Beijing, China) or TC assay kits (BIOSINO, Beijing, China) according to the manufacturers’ protocols.

### Histopathological examination

Fresh samples of liver and EAT were resected and fixed with 4% Paraformaldehyde at room temperature. The samples were dehydrated and embedded in paraffin, and then sectioned and further stained with hematoxylin-eosin staining (HE). Images were obtained using a LIOO 3.7 for digital camera software (Leica DM750, Leica Biosystems, Nussloch, Germany). Adipocytes size and distribution in EAT were measured and calculated from 5 fields per sample using 100 μm scale bar.

### 16S rRNA gene sequencing and analysis

Bacterial genomic DNA was extracted from cecal content or ileal content of animals with a QIAamp Fast DNA stool Mini Kit (51604, Qiagen, Germany) for subsequent 16S rRNA gene sequencing. Qualified DNA samples were purified and amplified using specific bacterial primers targeting the variable region V3–V4 of the 16S rRNA gene, and amplification primers were 341F (5′- CCTACGGGNBGCASCAG-3′) and 805R (5′- GACTACNVGGGTATCTAATCC-3′)^48^. Purified amplicons were pooled in equimolar and then paired-end sequencing was performed on an Illumina Hiseq 2500 sequencing platform at the Institute of Microbiology, Chinese Academy of Sciences (Beijing, China) according to standard protocols. The generated raw fastq files were quality-filtered and taxonomically analyzed using QIIME2 (V2019.7)^49^. In brief, primers of imported sequences were removed via Cutadapt (V2.4)^50^, and then DADA2 was used to filter and denoise sequences, remove chimeras, infer ASVs and generate the abundance table^51^. The ASVs were clustered with 100% similarity, and species annotation was performed for each ASV using the Naive Bayes classifier method based on the SILVA database^52^. For taxonomic annotation, all unassigned sequences and sequences annotated as mitochondria and chloroplasts were removed. Samples were rarefied to the same number of reads for the downstream analysis. Alpha diversity of each sample was assessed using the vegan package for R to assess bacterial richness and evenness. The weighted Unifrac based principal co-ordinates (PCoA) analysis was conducted to reflect differences in community structure of gut microbiota in each group, ANOSIM analysis was used to evaluate the statistical differences among groups. Differential bacteria analysis was assessed using Lefse (http://huttenhower.sph.harvard.edu/galaxy/). Levels of bacterial species showing statistically significant differences were evaluated using non-parametric factorial Kruskal–Wallis (KW) sum-rank test, and then Lefse uses linear discriminant analysis (LDA) scores to estimate the impact of each species abundance on the difference effect. The threshold of the logarithmic LDA score was 2.0. Correlation coefficients between bacterial genus or species and hepatic steatosis traits were determined using Spearman’s correlation analysis.

### Metagenomic shotgun sequencing of cecal microbiota

DNA in cecal content was extracted using a QIAamp Fast DNA stool Mini Kit (51604, Qiagen, Hilden, Germany), used agarose gel electrophoresis to test DNA integrity, Nanodrop to check DNA purity, and Qubit to quantify DNA concentration. Qualified DNA samples were randomly broken into fragments with a length of about 350 bp by an ultrasonic crusher, and sequencing library was prepared after end repair, adding A at 3’ end, adapter ligation, purification, fragment selection, and PCR amplification. The library was then subjected to quality inspection, and the library that passes the quality inspection is sequenced on an Illumina HiSeq platform at the Institute of Microbiology, Chinese Academy of Sciences (Beijing, China). Then, we filtered raw reads to obtain high-quality clean reads to ensure the accuracy of subsequent analysis results. Used SOAPdenovo2^53^ for assembly to obtain scaftigs, and useed MetaGeneMark^54^ for gene prediction based on effective Scaftig and structured a gene catalog, and then started from the gene catalog and integrated the CleanData of each sample to obtain the abundance information of the gene catalog in each sample. Follow-up analysis of the composition or difference of species between samples based on clean reads by using MetaPhlAn2 (v. 2.6.0) software^55^.

### Transcriptomics analysis of liver

The transcriptomics of liver tissue was performed by the Institute of Microbiology, Chinese Academy of Sciences (Beijing, China). Briefly, total RNA was extracted from liquid nitrogen-frozen liver tissue using by using TRIzol (Beyotime, Shanghai, China). The integrity of RNA samples was determined by Agilent 2100, and purity and concentration were detected by Nanodrop, to ensure the qualified samples were used for subsequent transcriptomic sequencing. Then, the library construction was carried out, and the main process is as follows: First, the eukaryotic mRNA was enriched with magnetic beads with Oligo (dT), and the mRNA was randomly interrupted by adding cation (Mg^2+^). Then, the mRNA was used as a template to synthesize successively first-strand cDNA and second-strand cDNA, and the double-stranded cDNA was purified using AMPure XP beads. After end repair, A-tailing and adapter ligation, the AMPure XP beads are used for fragment size selection. Finally, the final cDNA library was obtained by PCR enrichment. Next, the RNA-seq libraries were checked by using an Agilent 2100, and sequenced on Illumina HiSeq platform. Raw fastq files were used to calculate the amount of gene expression by RSEM software (http://deweylab.github.io/RSEM/). PCoA was used to present the relationship and variation between samples by dimensionality reduction of sample expression profiles. DESeq2, based on the negative binomial distribution, was used to analyze the raw counts to obtain the genes that compare the differences in expression between the groups (*p* adjust <0.05 & |log2FC|≥1). In this study, we used the R software package DESeq2 (version 1.32.0) for differential analysis to obtain the differential genes between different comparison groups and control group. Specifically, for the expression profile dataset, we removed genes with an expression value of 0 greater than 50%, and constructed an input matrix using the DESeqDataSetFromMatrix function. Then, we used the DESeq function and results function for data standardization and difference analysis, respectively. Finally, the difference significance of each gene was obtained. Additionally, gene ontology (GO, http://www.geneontology.org/) and Kyoto Encyclopedia of Genes and Genomes (KEGG, https://www.kegg.jp/kegg/rest/keggapi.html) enrichment analysis of the differential genes were carried out to determine the main biochemical metabolic pathway and signal transduction pathway involved in differentially expressed genes.

### Whole-genome sequencing

The DNA of *M. funiformis* CML154 was extracted using DNeasy Blood & Tissue Kit (Qiagen, Hilden, Germany). Sequencing library were constructed and sequenced on Illumina Hiseq 2500 sequencing platform at the Institute of Microbiology, Chinese Academy of Sciences (Beijing, China). Raw sequencing reads were quality trimmed using Trimmomatic v0.32^56^, and trimmed paired-end reads were assembled via de novo assembler IDBA-UD v1.1.1^57^. Filtered out contigs with coverage below 10% of average coverage of L50 contig coverage and the remaining contigs were scaffolded using SSPACE scaffolding v3.0^58^. Then, genomes are classified and annotated using eggNOG^59^ database and KEGG^60^ (Kyoto Encyclopedia of Genes and Genomes) database. Additionally, we constructed genome-scale metabolic modeling using gapseq and gapseq output was screened for a hand-curated set of pathways by using MetaCyc pathways^61^.

### Short chain fatty acids analysis

Targeted metabolomics was performed with GC-MS to quantify the concentrations of SCFAs in cecal content and bacterial fermentation broth. Briefly, for cecal content and bacterial fermentation broth samples, the extraction procedures have been carried out 4 °C to protect the volatile SCFAs. For cecal content samples, 50 mg cecal content were diluted with distilled water (4× the volume), tubes were homogenized and centrifuged at 12,000 × *g* for 15 min at 4 °C. Following centrifugation supernatants were transferred into centrifuge tubes for the derivatization reaction. 200 µL of 25% metaphosphoric acid solution was added to the supernatants, the solution was kept standing for 0.5 h with an environmental temperature of 4 °C, and then centrifuged at 12,000 × *g* for 10 min at 4 °C. Next, transfer the obtained supernatant to glass bottles for assay. the concentration gradient of standard curve was 0–100 μg/mL for low concentration and 0–600 μg/mL for high concentration. SCFAs levels in the cecal content were normalized to the weight. For bacterial fermentation broth samples, 2 mL bacterial fermentation broth was centrifuged at 12,000 × *g* for 10 min at 4 °C, and obtained the supernatant, and subsequent processing steps was consistent with cecal content samples.

### Real-time quantitative PCR

Total RNA from tissues or cells were isolated using the TRIzol method, and then used to synthesize the first-strand cDNA cDNA with the PrimeScript ^TM^ RT reagent Kit with gDNA Eraser (RR047A, TaKaRa, Japan). Real-time qPCR was carried out using SYBR^®^ Premix Ex TaqTM (RR420, TaKaRa, Japan), 384-well plates and the QuantStudio^TM^ 7 Flex Real-Time PCR System (Thermo Fisher Scientific, USA). The results were analyzed using the 2^−ΔΔCt^ method with β-actin serving as the reference. The primers used were synthesized by Sangon Biotech (Shanghai, China), and listed in Supplementary Table 11.

### Statistical analysis

Statistical analysis was performed using GraphPad Prism version 8.0 (GraphPad Software, San Diego, CA, USA) and data are shown as means ± SEM. Significant differences between the two groups were assessed by unpaired two-tailed Student’s *t* test or Mann–Whitney U test for samples that were not normally distributed. Data sets involving more than two groups were evaluated by one-way analysis of variance (ANOVA) followed by the Least Significant Difference (LSD) or by the non-parametric Kruskal–Wallis test with IBM SPSS Statistics 24.0 (SPSS Inc., Chicago, IL, USA). *P* < 0.05 was considered to be statistically significant.

### Reporting summary

Further information on research design is available in the Nature Research Reporting Summary linked to this article.

### Supplementary information

Supplementary Figures

Supplementary Tables

Reporting Summary

## Data Availability

The datasets generated for this study can be found in the Sequence Read Archive of the NCBI (Accession numbers PRJNA831016, PRJNA909010, PRJNA908890, PRJNA908125, PRJNA902159).
