# Author Correction: Inulin-enriched Megamonas funiformis ameliorates metabolic dysfunction-associated fatty liver disease by producing propionic acid

**DOI:** 10.1038/s41522-024-00480-1

**Published:** 2024-01-29

**Authors:** Xinyue Yang, Meihong Zhang, Yan Liu, Fuxiao Wei, Xin Li, Yuqing Feng, Xiaolu Jin, Dan Liu, Yuming Guo, Yongfei Hu

**Affiliations:** State Key Laboratory of Animal Nutrition and Feeding, College of Animal Science and Technology, China Agricultural University, 100193 Beijing, China

**Keywords:** Microbiome, Metagenomics

Correction to: *npj Biofilms and Microbiomes* 10.1038/s41522-023-00451-y, published online 4 November 2023

In this article, incorrect text was inserted in the “Results” section “*M. funiformis* CML154 prevents diet-induced MAFLD in laying hen model”.

The incorrect text, “We then explored whether the effect of *M. funiformis* CML154 in alleviating MAFLD is host species-specific. Given mice are the most commonly used animal model for studying fatty liver disease, male C57BL/6J mice were maintained on either ND or HFD with or without oral gavage of *M. funiformis* CML154 for 9 weeks.” has been corrected to “To investigate whether *M. funiformis* CML154 has anti-MAFLD effect, hens were maintained on either normal chow diet or HFD with or without oral gavage of *M. funiformis* CML154 for 19 weeks.” in the original article.

