# Supplementary material for: Inulin-enriched Megamonas funiformis ameliorates metabolic dysfunction-associated fatty liver disease by producing propionic acid
Source: NPJ Biofilms Microbiomes. 2023 Nov 4;9:84. doi: 10.1038/s41522-023-00451-y (PMC10625582; doi:10.1038/s41522-023-00451-y)
Supplement: Supplementary file 1 — Supplementary Figures [file 41522_2023_451_MOESM1_ESM.pdf]

# Inulin-enriched *Megamonas funiformis* ameliorates metabolic dysfunction-associated fatty liver disease by producing propionic acid

Xinyue Yang[1], Meihong Zhang[1], Yan Liu[1], Fuxiao Wei[1], Xin Li[1], Yuqing Feng[1], Xiaolu Jin[1], Dan Liu[1], Yuming Guo[1], Yongfei Hu[1]*

[1]State Key Laboratory of Animal Nutrition and Feeding, College of Animal Science and Technology, China Agricultural University, Beijing 100193, China

*Correspondence to Yongfei Hu, huyongfei@cau.edu.cn; Tel.: +86-10-62732225.

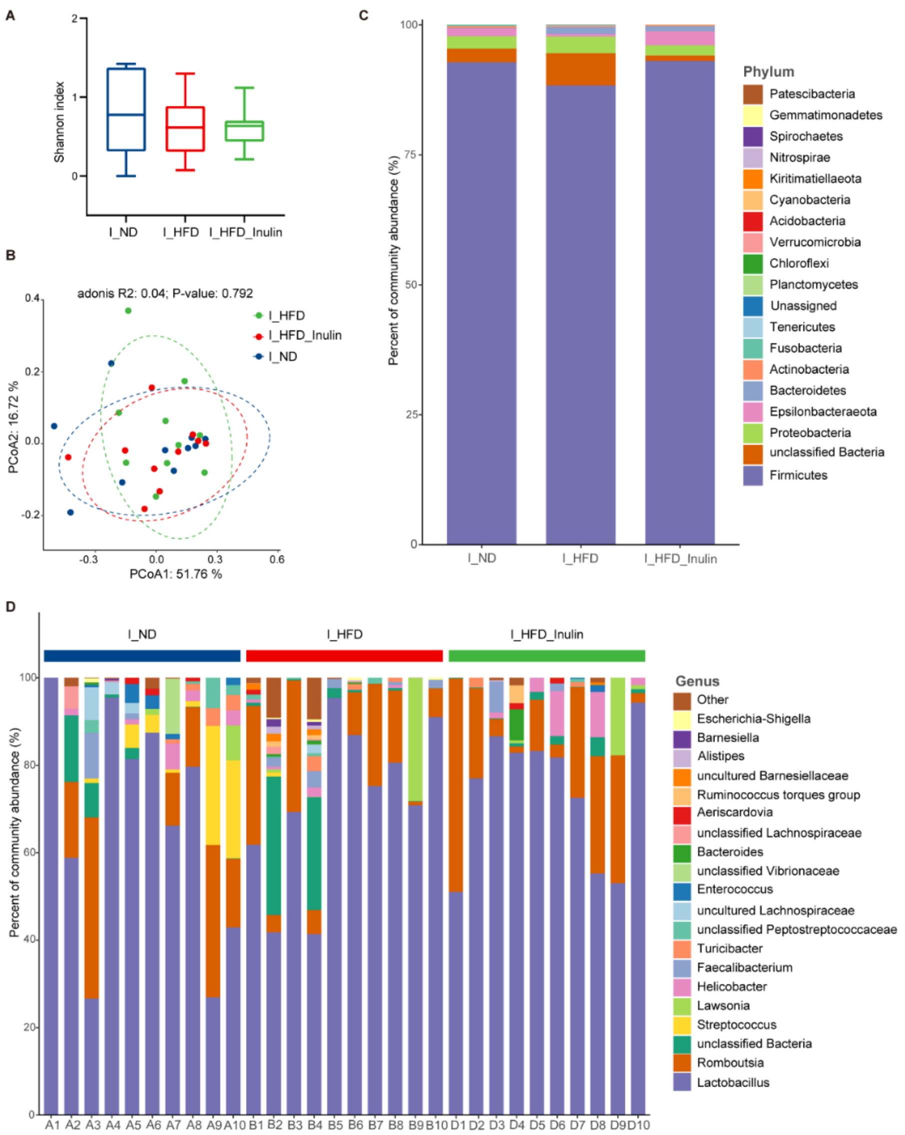


**Supplementary Fig. 1. Inulin does not alter ileal microbial diversity and composition of HFD-induced MAFLD laying hens.** (A) Alpha diversity of ileal microbiota in different groups. Data were analyzed by nonparametric Kruskal-Wallis test. (B) Principal coordinate analysis (PCoA) of

ileal microbiota in different groups. PCoA plots were generated using ASV abundance data according to the Bray–Curtis distance, and statistical significance was measured using adonis analysis. (C) Relative abundance of bacteria at the phylum level. (D) Relative abundance of top 20 genera in each sample. *n* = 10 hens per group.

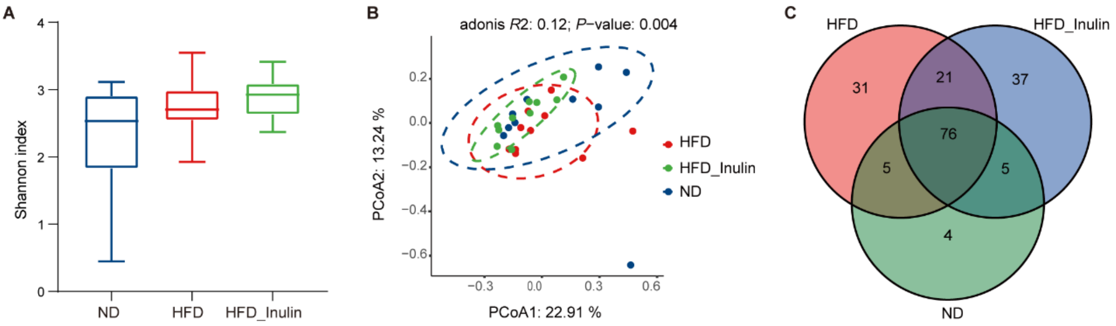


**Supplementary Fig. 2. Effects of inulin on cecal microbiota diversity in HFD-induced MAFLD laying hens.** (A) The alpha diversity of cecal microbiota in different groups. Data were analyzed by nonparametric Kruskal-Wallis test. (B) Principal coordinate analysis (PCoA) of cecal microbiota in different groups. PCoA plots were generated using ASV abundance data according to the Bray–Curtis distance, and statistical significance was measured using adonis analysis. (C) The Venn diagram shows the shared microbes among different groups. *n* = 10 hens per group.

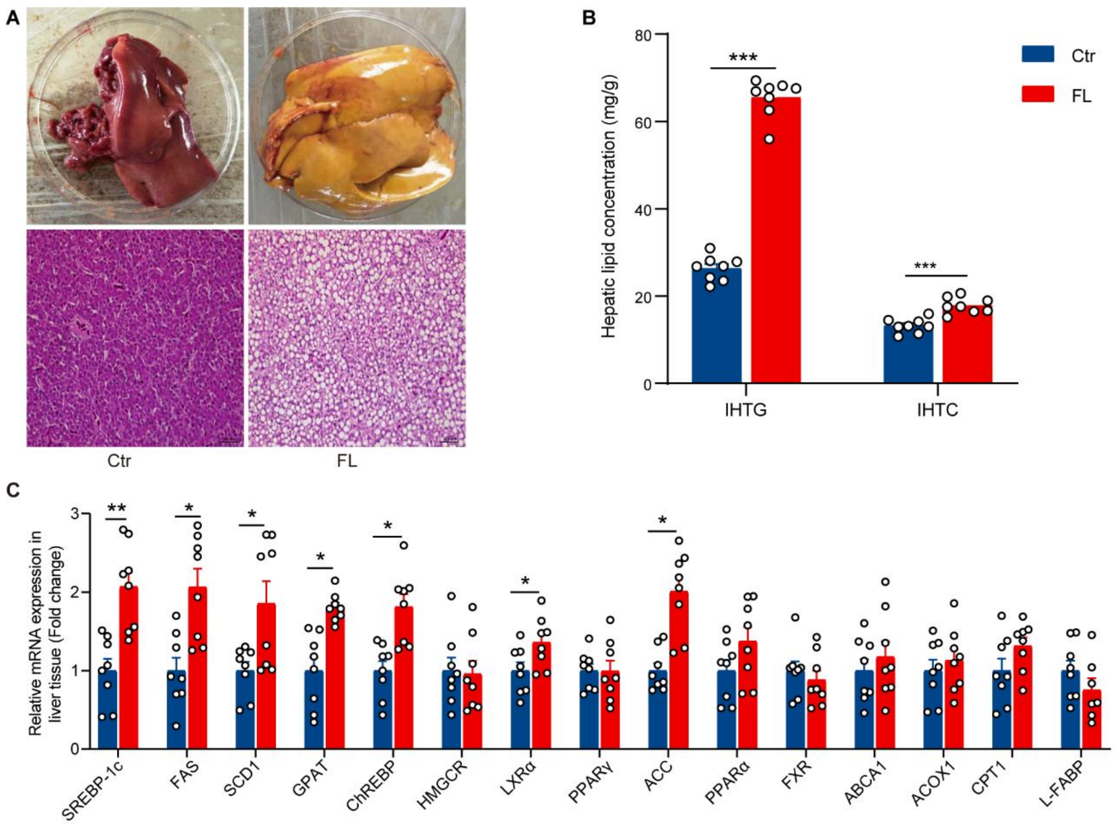


**Supplementary Fig. 3. Characteristics of hepatic steatosis in healthy and fatty liver old laying hens**. (A) Representative photomicrographs of liver tissues with H&E staining (Scale bars = 200 μm) and phenotype pictures. (B) Hepatic lipid levels (mg/g liver). (C) The relative mRNA expression of lipid metabolism related genes in liver tissues. Data are presented as the mean ± SEM; *n* = 7-8 hens per group. Statistical analysis was performed using independent-samples *t* test. *$P <$ 0 .05, **$P < 0.01$, ***$P < 0.001$.

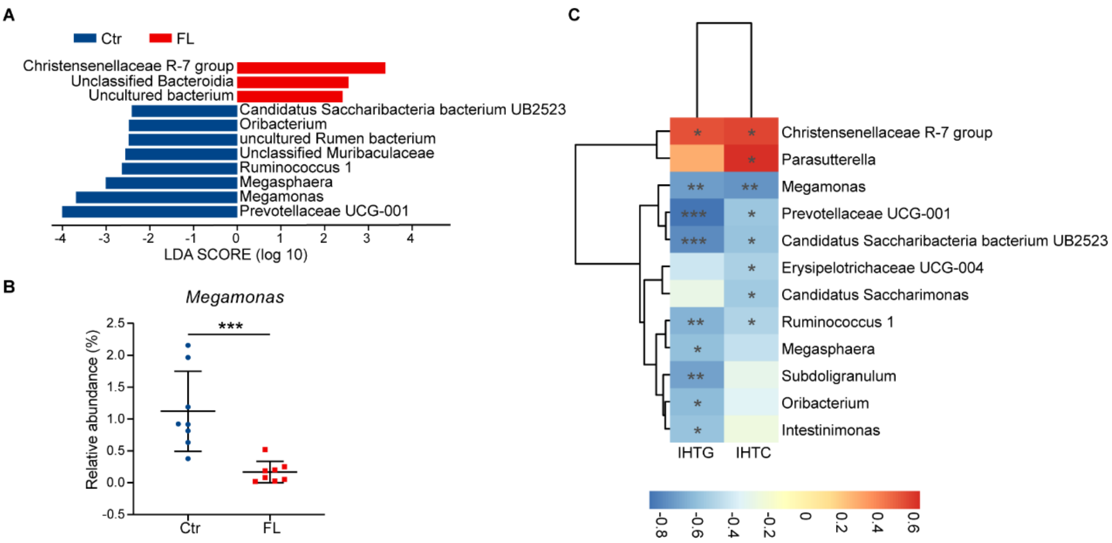


**Supplementary Fig. 4. Differentially abundant microbes in healthy and fatty liver old laying hens.** (A) Differences in the bacterial communities between different groups at the genus level. Significant differences in Linear discriminant analysis (LDA) scores ($P < 0.05$) were produced between groups (Wilcoxon's test). The threshold of the logarithmic LDA score was 2.0. (B) Relative abundance of *Megamonas*. Data were analyzed using non-parametric Mann-Whitney U test. (C) The Spearman's rank correlation analysis between intrahepatic lipid content and cecal microbes at the genus level: *$P < 0$ .05, **$P < 0.01$, ***$P < 0.001$. $n = 8$ hens per group.

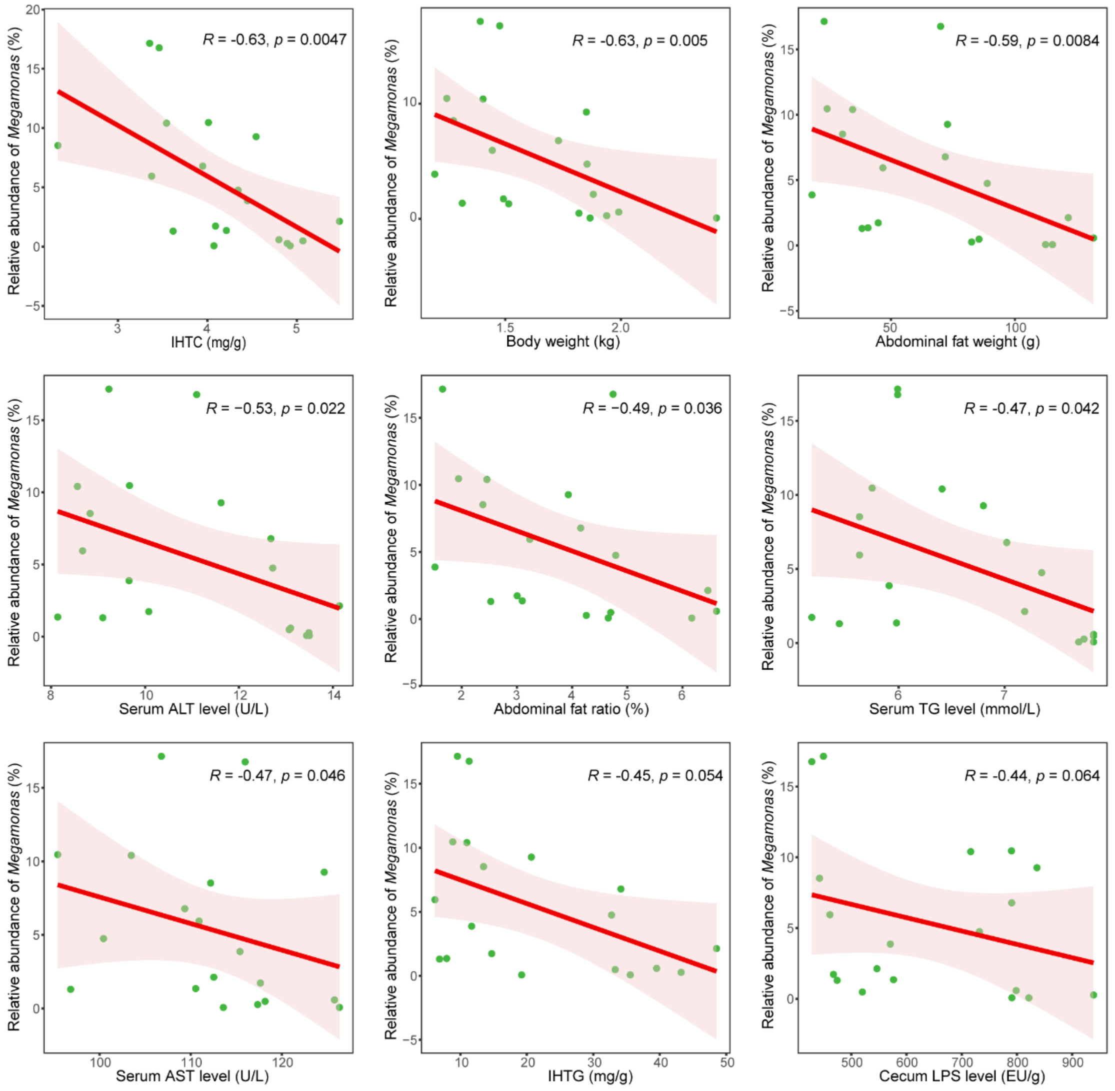


**Supplementary Fig. 5. Spearman's correlation analysis between the relative abundance of inulin-enriched *Megamonas* and hepatic steatosis traits based on metagenomic data.** Lines represent the linear model fit. R, correlation coefficient. *n* = 10 hens per group. Samples with non-detectable levels of *Megamonas* were not included.

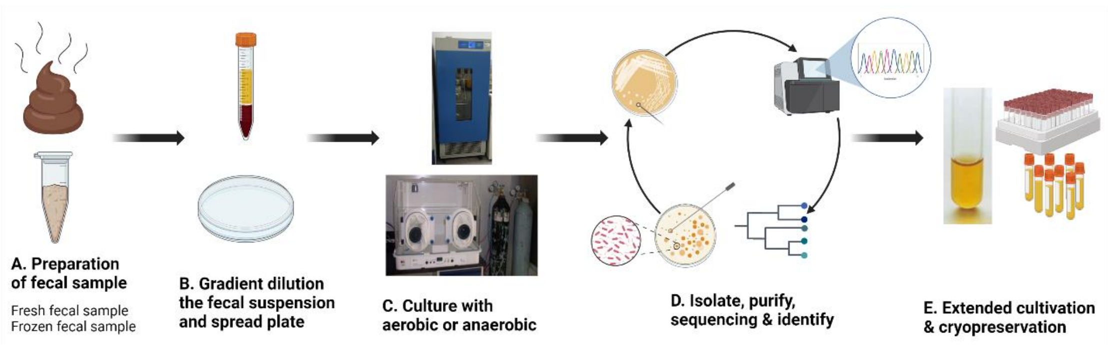

**Supplementary Fig. 6. A workflow for culturomics.** (A) Fresh or frozen cecal contents of HFD_Inulin group hens were obtained and then homogenized into fecal suspension. (B) The fecal suspension was serially diluted and spread on different media agar plates, (C) and then placed in the aerobic or anaerobic incubator for constant temperature cultivation at 37 °C. (D) Select single colony for purification, full-length16S rRNA sequencing and sequence alignment to identify the isolates. (E) Each isolate was expanded and cryopreserved.

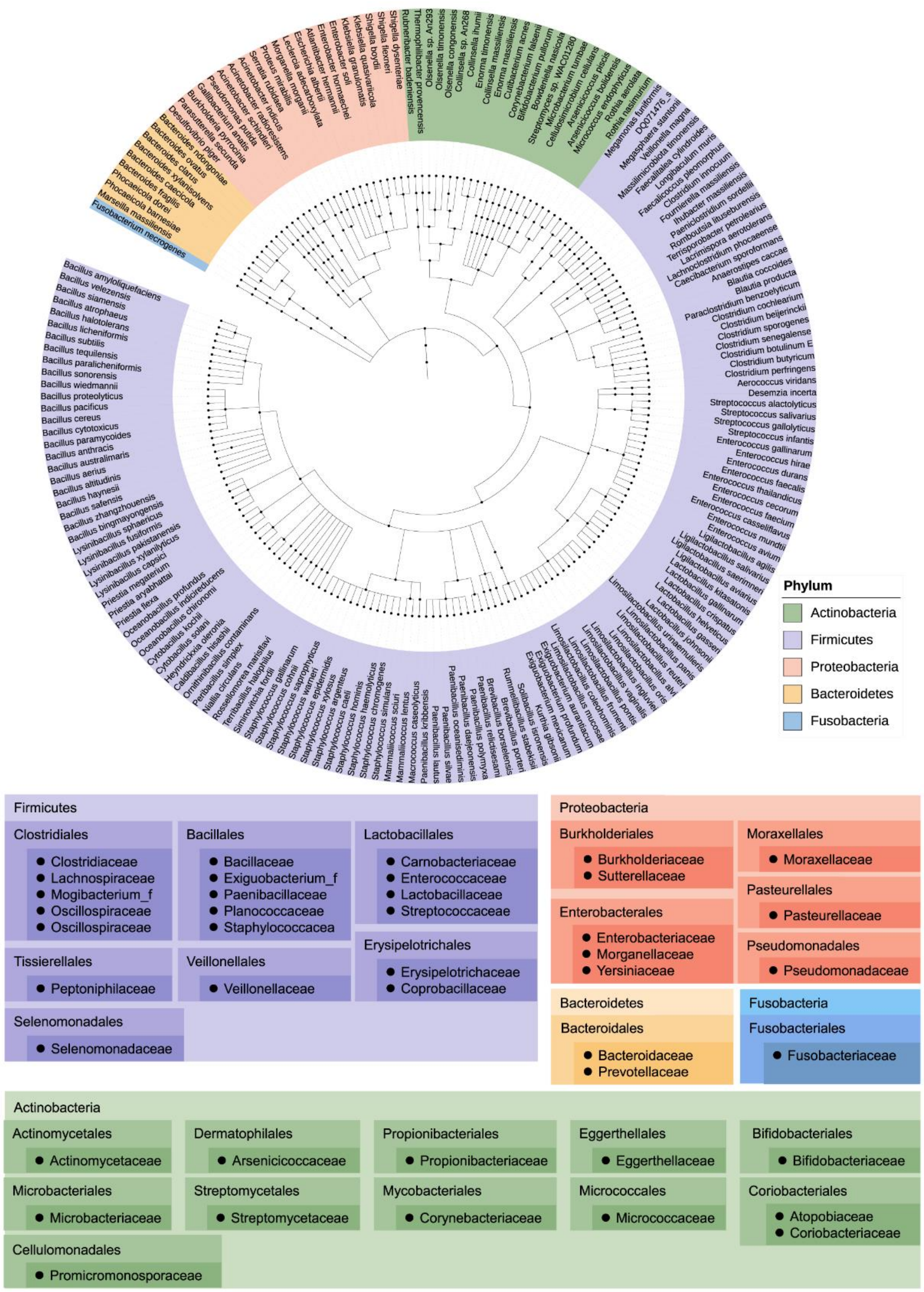


**Supplementary Fig. 7. Phylogenetic tree of the 193 species isolated by culturomics.** Phylogenetic tree was constructed from the 16S rRNA gene sequences of hen microbiota members at the species level. The tree is color coded according to the phyla. Bacterial species from Firmicutes, Actinobacteria, Proteobacteria, Bacteroidetes, and Fusobacteria are highlighted in purple, green, red, orange, and blue, respectively. The tree was generated using MEGA 11 and decorated by iTOL.

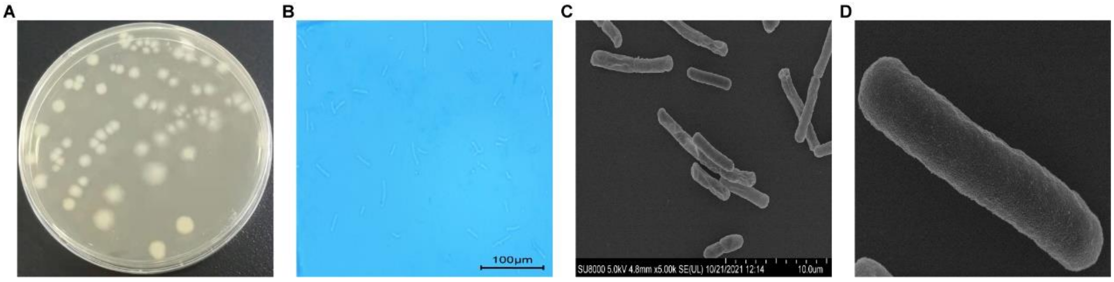


**Supplementary Fig. 8. Morphology of strain *M. funiformis* CML154.** (A) Colony morphology grown on GAM agar plate. (B) Light micrographs of cells grown in GAM broth (Scale bars = 100 μm). (C–D) Scanning electron micrographs of cells grown in GAM broth. Bar, 10 μm (C) & 2 μm (D).

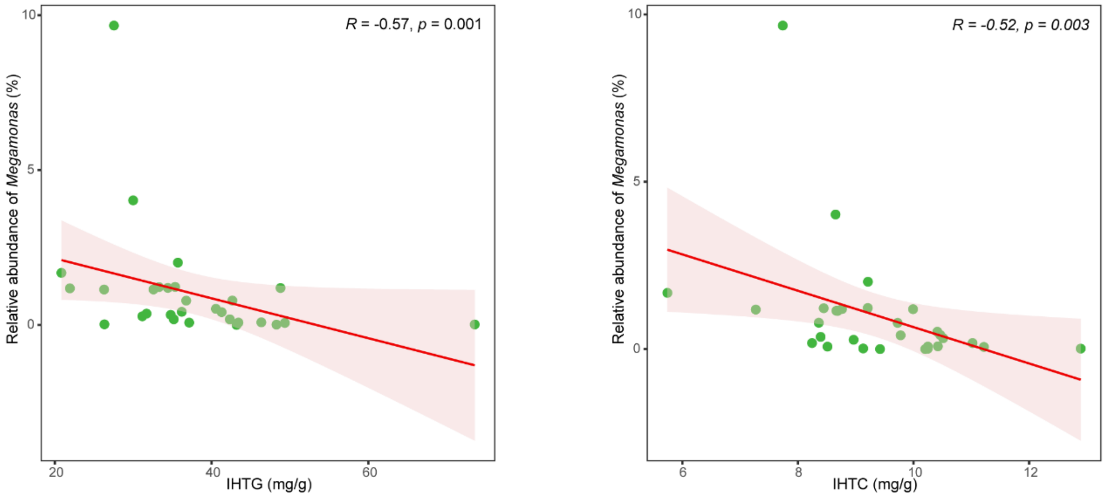


**Supplementary Fig. 9. Spearman's correlation analysis between the relative abundance of *Megamonas* and intrahepatic lipid content in *M. funiformis* CML154-gavaged laying hens.** Lines represent the linear model fit. *R*, correlation coefficient, *n* = 10 hens per group.

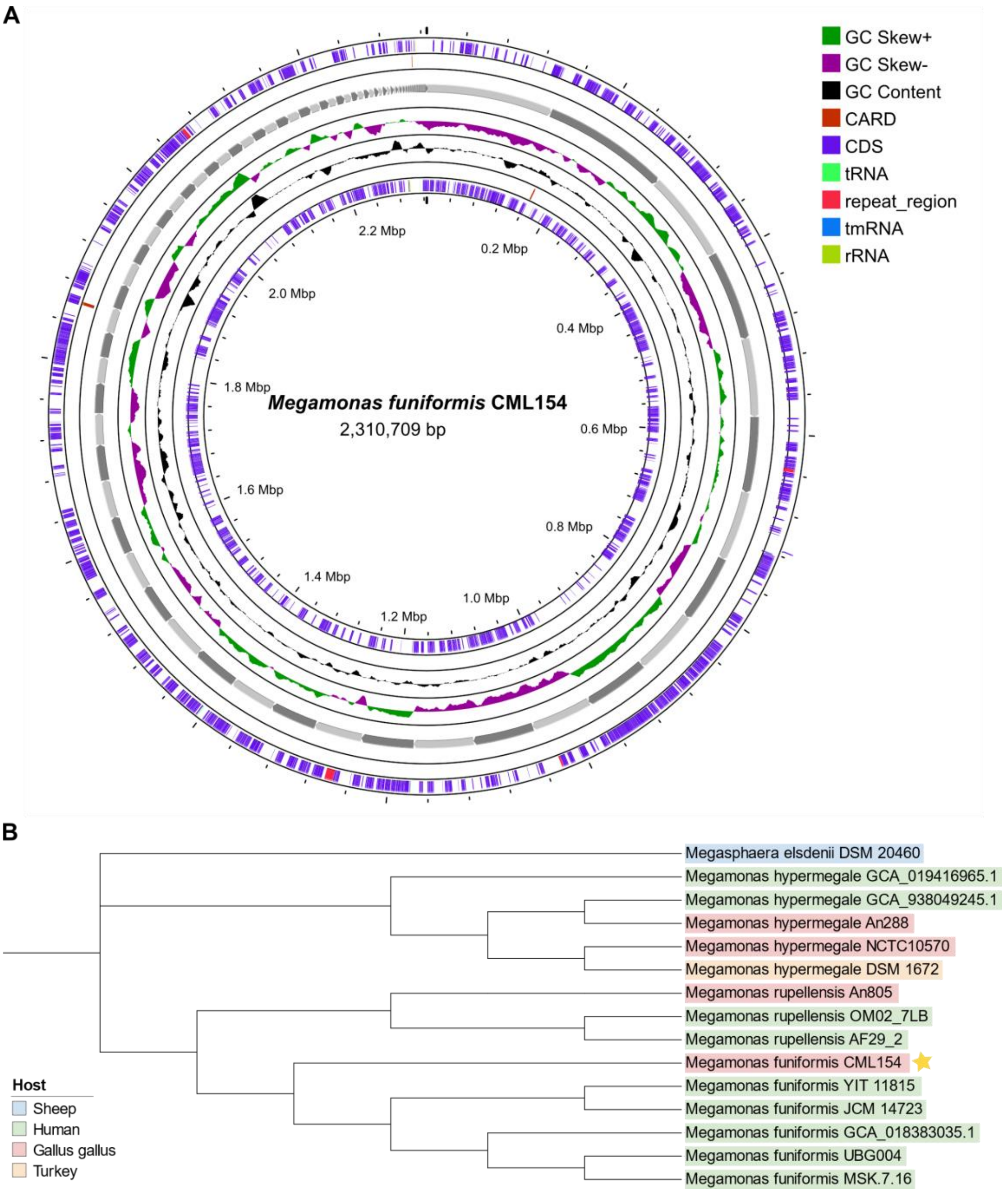


**Supplementary Fig. 10. Genomic and phylogenetic analysis of *M. funiformis* CML154.** (A) The genome map of *M. funiformis* CML154. (B) The phylogenetic tree of *M. funiformis* CML154 isolates and different *Megamonas* species with different origins. The phylogenetic tree was constructed by adding *Megasphaera elsdenii* DSM 20460 as an outgroup based on maximum likelihood method. ☆ represents *M. funiformis* CML154.

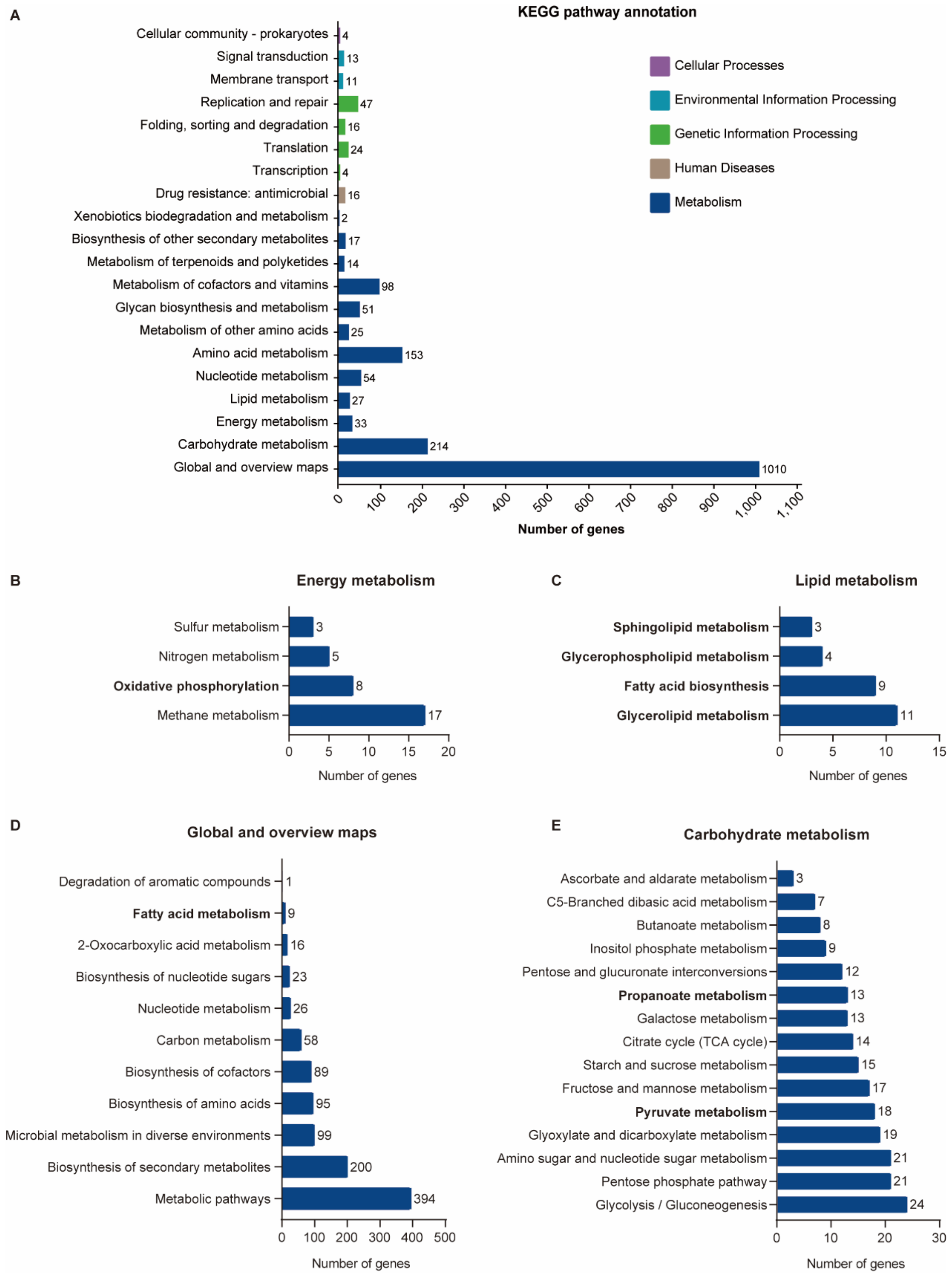


**Supplementary Fig. 11. KEGG pathway annotation of *M. funiformis* CML154.** KEGG pathways of *M. funiformis* CML154 in five major classes (A), and in four subclasses (B-E).

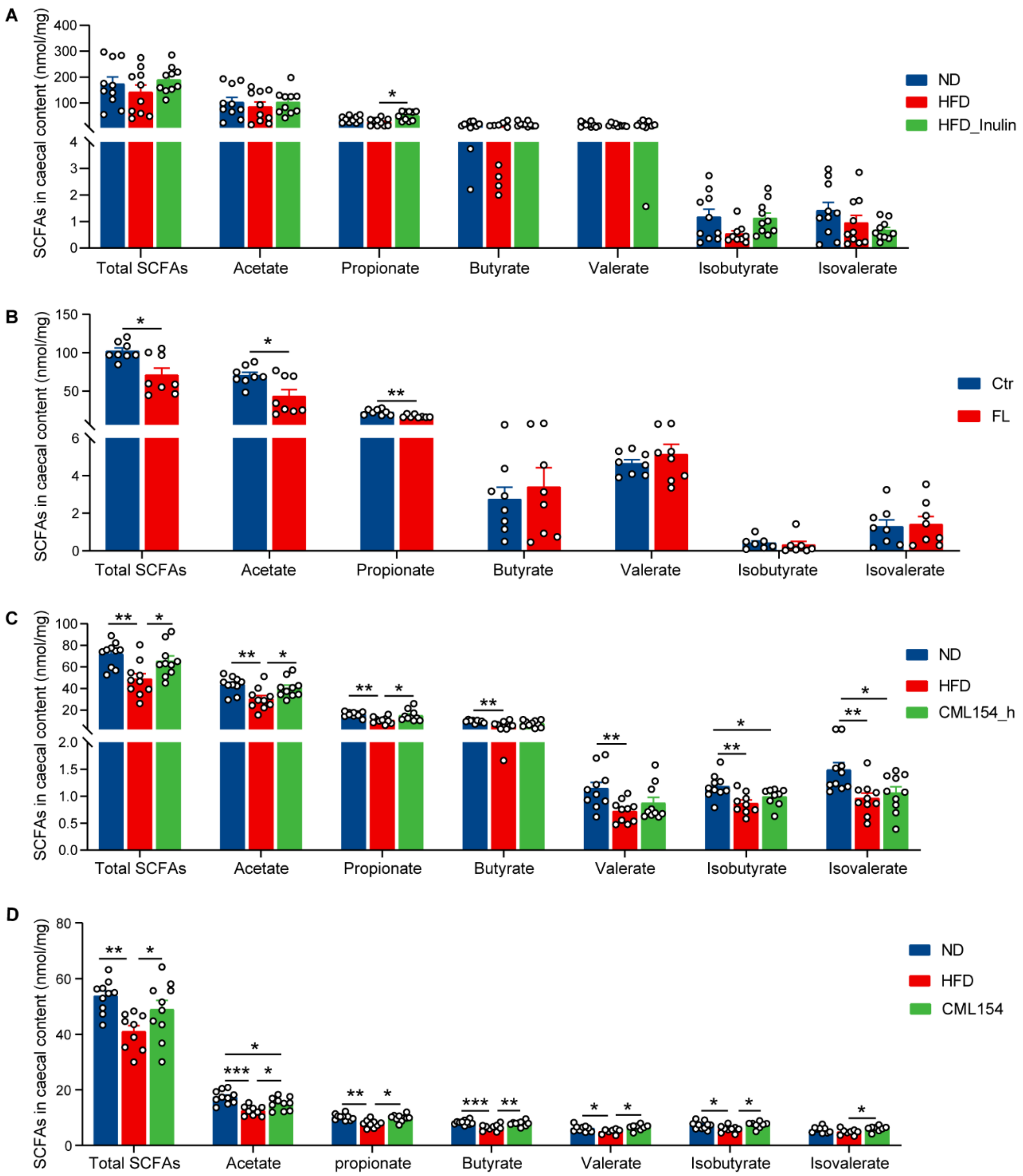


**Supplementary Fig. 12. Targeted metabolomic analyses of SCFA levels using GC-MS in *in vivo* experiments.** (A) SCFA concentrations in the cecal contents of inulin-treated laying hens ($n$ = 9-10 hens per group). (B) SCFA concentrations in the cecal contents of old laying hens ($n$ = 7-8 hens per group). (C) SCFA concentrations in the cecal contents of *M. funiformis* CML154-gavaged laying hens ($n$ = 10 hens per group). (D) SCFA concentrations in the cecal content of *M. funiformis* CML154-gavaged mice ($n$ = 7-10 mice per group). Data are presented as the mean ± SEM. Statistical analysis was performed using one-way ANOVA followed by the LSD or independent-

samples *t* test. *$P < 0.05$, **$P < 0.01$, ***$P < 0.001$.

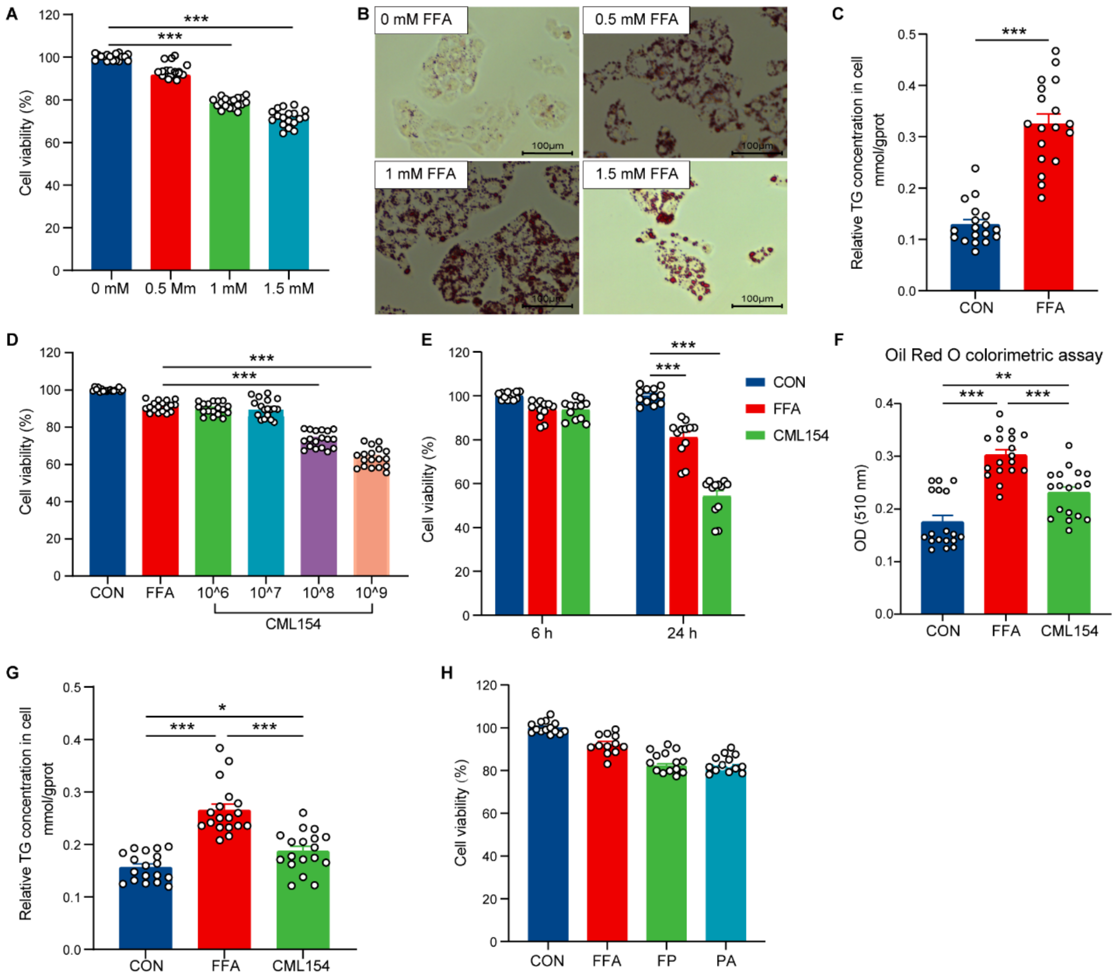


**Supplementary Fig. 13. Exploring the conditions of cell treatment by live *M. funiformis* CML154 (CML154), its fermentation products (FP) and propionate (PA).** (A) Cell viability under stimulation with different concentrations of FFA. (B) Representative photomicrographs of hepatocytes with Oil red O staining (Scale bars = 100 μm). (C) TG level in hepatocytes. (D) Cell viability stimulated by different concentrations of live CML154. (E) Cell viability under stimulation with live CML154 for 6 hours and 24 hours, respectively. (F) Oil red O colorimetric assay. (G) TG level in hepatocytes. (H) Cell viability under different concentrations of metabolites and propionate. $n = 6$ wells per group, and each experiment was repeated three times. Statistical analysis was performed using one-way ANOVA followed by the LSD. *$P < 0\ .05$, **$P < 0.01$, ***$P < 0.001$.

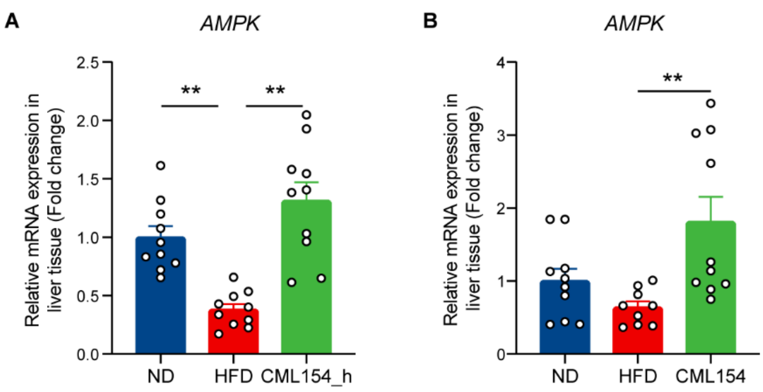


**Supplementary Fig. 14.** The relative mRNA expression of *AMPK* in liver of *M. funiformis* CML154-gavaged laying hens (A) ($n$ = 10), and *M. funiformis* CML154-gavaged mice (B) ($n$ = 9-10). Statistical analysis was performed using the non-parametric Kruskal-Wallis test. $^{**}P < 0.01$.
