# Supplementary material for: Inulin-enriched Megamonas funiformis ameliorates metabolic dysfunction-associated fatty liver disease by producing propionic acid
Source: NPJ Biofilms Microbiomes. 2023 Nov 4;9:84. doi: 10.1038/s41522-023-00451-y (PMC10625582; doi:10.1038/s41522-023-00451-y)
Supplement: Supplementary file 3 — Reporting Summary [file 41522_2023_451_MOESM3_ESM.pdf]

nature portfolio

Corresponding author(s): Yongfei Hu
Last updated by author(s): 2023-4-7

Reporting Summary

Nature Portfolio wishes to improve the reproducibility of the work that we publish. This form provides structure for consistency and transparency in reporting. For further information on Nature Portfolio policies, see our Editorial Policies and the Editorial Policy Checklist.

Statistics

For all statistical analyses, confirm that the following items are present in the figure legend, table legend, main text, or Methods section.

n/a	Confirmed	
☐	☒	The exact sample size (*n*) for each experimental group/condition, given as a discrete number and unit of measurement
☐	☒	A statement on whether measurements were taken from distinct samples or whether the same sample was measured repeatedly
☐	☒	The statistical test(s) used AND whether they are one- or two-sided<br>*Only common tests should be described solely by name; describe more complex techniques in the Methods section.*
☐	☒	A description of all covariates tested
☐	☒	A description of any assumptions or corrections, such as tests of normality and adjustment for multiple comparisons
☐	☒	A full description of the statistical parameters including central tendency (e.g. means) or other basic estimates (e.g. regression coefficient) AND variation (e.g. standard deviation) or associated estimates of uncertainty (e.g. confidence intervals)
☐	☒	For null hypothesis testing, the test statistic (e.g. *F*, *t*, *r*) with confidence intervals, effect sizes, degrees of freedom and *P* value noted<br>*Give P values as exact values whenever suitable.*
☐	☒	For Bayesian analysis, information on the choice of priors and Markov chain Monte Carlo settings
☐	☒	For hierarchical and complex designs, identification of the appropriate level for tests and full reporting of outcomes
☐	☒	Estimates of effect sizes (e.g. Cohen's *d*, Pearson's *r*), indicating how they were calculated

Our web collection on statistics for biologists contains articles on many of the points above.

Software and code

Policy information about availability of computer code

Data collection	*Provide a description of all commercial, open source and custom code used to collect the data in this study, specifying the version used OR state that no software was used.*
Data analysis	*Provide a description of all commercial, open source and custom code used to analyse the data in this study, specifying the version used OR state that no software was used.*

For manuscripts utilizing custom algorithms or software that are central to the research but not yet described in published literature, software must be made available to editors and reviewers. We strongly encourage code deposition in a community repository (e.g. GitHub). See the Nature Portfolio guidelines for submitting code & software for further information.

Data

Policy information about availability of data

All manuscripts must include a data availability statement. This statement should provide the following information, where applicable:

- Accession codes, unique identifiers, or web links for publicly available datasets
- A description of any restrictions on data availability
- For clinical datasets or third party data, please ensure that the statement adheres to our policy

The datasets generated for this study can be found in the Sequence Read Archive of the NCBI (Accession numbers PRJNA831016, PRJNA909010, PRJNA908890, PRJNA908125, PRJNA902159).

Research involving human participants, their data, or biological material

Policy information about studies with human participants or human data. See also policy information about sex, gender (identity/presentation), and sexual orientation and race, ethnicity and racism.

Reporting on sex and gender	*Use the terms sex (biological attribute) and gender (shaped by social and cultural circumstances) carefully in order to avoid confusing both terms. Indicate if findings apply to only one sex or gender; describe whether sex and gender were considered in study design; whether sex and/or gender was determined based on self-reporting or assigned and methods used. Provide in the source data disaggregated sex and gender data, where this information has been collected, and if consent has been obtained for sharing of individual-level data; provide overall numbers in this Reporting Summary. Please state if this information has not been collected. Report sex- and gender-based analyses where performed, justify reasons for lack of sex- and gender-based analysis.*
Reporting on race, ethnicity, or other socially relevant groupings	*Please specify the socially constructed or socially relevant categorization variable(s) used in your manuscript and explain why they were used. Please note that such variables should not be used as proxies for other socially constructed/relevant variables (for example, race/ethnicity should not be used as a proxy for socioeconomic status). Provide clear definitions of the relevant terms used, how they were provided (by the participants/respondents, the researchers, or third parties), and the method(s) used to classify people into the different categories (e.g. self-report, census or administrative data, social media data, etc.) Please provide details about how you controlled for confounding variables in your analyses.*
Population characteristics	*Describe the covariate-relevant population characteristics of the human research participants (e.g. age, genotypic information, past and current diagnosis and treatment categories). If you filled out the behavioural & social sciences study design questions and have nothing to add here, write "See above."*
Recruitment	*Describe how participants were recruited. Outline any potential self-selection bias or other biases that may be present and how these are likely to impact results.*
Ethics oversight	*Identify the organization(s) that approved the study protocol.*

Note that full information on the approval of the study protocol must also be provided in the manuscript.

Field-specific reporting

Please select the one below that is the best fit for your research. If you are not sure, read the appropriate sections before making your selection.

☒ Life sciences ☐ Behavioural & social sciences ☐ Ecological, evolutionary & environmental sciences

For a reference copy of the document with all sections, see nature.com/documents/nr-reporting-summary-flat.pdf

Life sciences study design

All studies must disclose on these points even when the disclosure is negative.

Sample size	We describe the sample size in detail in the manuscript, and the sample size is based on the number of samples in each treatment group not less than 6.
Data exclusions	In this study, we removed individual anomalous data using Grubbs removal criteria.
Replication	In order to verify the anti-MAFLD effect of Megamonas, we used two animal models, mouse and hen, both of which confirmed the anti-MAFLD effect of Megamonas. In addition, in cell experiments, each of our results was determined by repeating three times.
Randomization	All experiments involved in this study randomly assigned their samples to different experimental groups as we indicated in the manuscript.
Blinding	Blinding was not possible in this study as the purpose of our study was to compare the results under different interventions.

Reporting for specific materials, systems and methods

We require information from authors about some types of materials, experimental systems and methods used in many studies. Here, indicate whether each material, system or method listed is relevant to your study. If you are not sure if a list item applies to your research, read the appropriate section before selecting a response.

Materials & experimental systems

n/a	Involved in the study
☐	☒ Antibodies
☐	☒ Eukaryotic cell lines
☒	☐ Palaeontology and archaeology
☐	☒ Animals and other organisms
☒	☐ Clinical data
☒	☐ Dual use research of concern
☒	☐ Plants

Methods

n/a	Involved in the study
☒	☐ ChIP-seq
☒	☐ Flow cytometry
☒	☐ MRI-based neuroimaging

Antibodies

Antibodies used

neomycin (1 g/L; 7177–48-2, Sigma, USA), ampicillin (1 g/L; 7177–48-2, Sigma, USA), metronidazole (1 g/L; 443–48-1, Sigma, USA) and vancomycin (0.5 g/L; 1404–90-6, Sigma, USA)

Validation

We used an antibiotic cocktail (ampicillin, vancomycin, neomycin, and metronidazole) before FMT with the aim of reducing the microbial load in the gut, which is a common approach in FMT studies.REF:[1] Sarrabayrouse G, et al. Mucosal microbial load in Crohn's disease: A potential predictor of response to faecal microbiota transplantation. EBioMedicine 51, 102611 (2020).[2] Hintze KJ, et al. Broad scope method for creating humanized animal models for animal health and disease research through antibiotic treatment and human fecal transfer. Gut Microbes 5, 183-141 (2014).[3] Turnbaugh PJ, Ridaura VK, Faith JJ, Rey FE, Knight R, Gordon JI. The effect of diet on the human gut microbiome: a metagenomic analysis in humanized gnotobiotic mice. Sci Transl Med 1, 6ra14 (2009).[4] Staley C, et al. Stable engraftment of human microbiota into mice with a single oral gavage following antibiotic conditioning. Microbiome 5, 87 (2017).

Eukaryotic cell lines

Policy information about cell lines and Sex and Gender in Research

Cell line source(s)

Human

Authentication

The HepG2 cell line used in this study passed STR identification.

Mycoplasma contamination

We confirmed that the HepG2 cell line tested negative for mycoplasma contamination.

Commonly misidentified lines (See ICLAC register)

None

Animals and other research organisms

Policy information about studies involving animals; ARRIVE guidelines recommended for reporting animal research, and Sex and Gender in Research

Laboratory animals

24-week-old Hy-Line grey commercial laying hens; 85-week-old Hy-Line brown commercial laying hens; 6-week-old C57BL/6J male mice

Wild animals

This study did not involve wild animals.

Reporting on sex

Hens and male mice were used as laboratory animals in this study, and our findings were not affected by gender.

Field-collected samples

All laying hens were housed in Zhuozhou breeding base of China Agricultural University (Zhuozhou, China). All hens were housed in pairs in stainless-steel ladder cages (45×45×45 cm) and in a controlled room, in which temperature and relative humidity was maintained at 26 ± 2°C and 50 ± 10%, respectively. The photoperiod was a 16 h light/8 h dark with a dark cycle encompassing 10 pm to 6 am. All the birds were allowed free access to water and food. During the experiments, food intake was recorded once a week. All mice were housed in SPF (specific pathogen free) conditions in Auxiliary building of teaching center of College of Biology (China Agricultural University, Beijing, China). Mice were housed in stainless steel cages (five mice/cage) with free access to sterile water and sterile food (irradiated), and kept in a controlled environment (room temperature maintained at 25 ± 1°C, 12 h dark-light cycle with a dark cycle encompassing 8 p.m. to 8 a.m.).

Ethics oversight

All animal experimental procedures were conducted according to guidelines of the Institutional Studies Animal Care and Use Committee of China Agricultural University (Beijing, China). The study was approved by the Ethics Committees of Laboratory Animal Center of China Agricultural University (Beijing, China) (permit AW13501202-2-2).

Note that full information on the approval of the study protocol must also be provided in the manuscript.
